# The lymphatic system favours survival of a unique *T. brucei* population

**DOI:** 10.1242/bio.059992

**Published:** 2023-11-09

**Authors:** Henrique Machado, António Temudo, Mariana De Niz

**Affiliations:** ^1^ Instituto de Medicina Molecular João Lobo Antunes, Faculdade de Medicina, Universidade de Lisboa, Lisboa 1649-028, Portugal; ^2^ Bioimaging Unit, Instituto de Medicina Molecular João Lobo Antunes, Faculdade de Medicina, Universidade de Lisboa, Lisboa 1649-028, Portugal

**Keywords:** Lymph nodes, Lymphatic system, Parasitology, *Trypanosoma brucei*

## Abstract

*Trypanosoma brucei* colonise and multiply in the blood vasculature, as well as in various organs of the host's body. Lymph nodes have been previously shown to harbour large numbers of parasites, and the lymphatic system has been proposed as a key site that allows *T. brucei* distribution through, and colonization of the mammalian body. However, visualization of host-pathogen interactions in the lymphatic system has never captured dynamic events with high spatial and temporal resolution throughout infection. In our work, we used a mixture of tools including intravital microscopy and *ex vivo* imaging to study *T. brucei* distribution in 20 sets of lymph nodes. We demonstrate that lymph node colonization by *T. brucei* is different across lymph node sets, with the most heavily colonised being the draining lymph nodes of main tissue reservoirs: the gonadal white adipose tissue and pancreas. Moreover, we show that the lymphatic vasculature is a pivotal site for parasite dispersal, and altering this colonization by blocking LYVE-1 is detrimental for parasite survival. Additionally, parasites within the lymphatic vasculature have unique morphological and behavioural characteristics, different to those found in the blood, demonstrating that across both types of vasculature, these environments are physically separated. Finally, we demonstrate that the lymph nodes and the lymphatic vasculature undergo significant alterations during *T. brucei* infection, resulting in oedema throughout the host's body.

## INTRODUCTION

*Trypanosoma brucei* is an extracellular parasite transmitted by the bite of infected tsetse flies (*Glossina* spp.). These parasites are known to colonise and multiply in the blood vasculature, as well as in the extravascular space of several organs (reviewed in Crilly and Mugnier, 2021; Silva Pereira et al., 2019). Historical studies have shown that *T. brucei* parasites have a preference for the lymphatic system (Goodwin, 1970; Schuberg and Boeing, 1913). While we and others have identified large *T. brucei* chronic reservoirs in tissues including the gonadal white adipose tissue (g-WAT) (De Niz et al., 2021; Trindade et al., 2022, 2016), pancreas (De Niz et al., 2021), brain (Coles et al., 2015; Myburgh et al., 2013), skin (Alfituri et al., 2020a; Capewell et al., 2016) and lungs (Mabille et al., 2022), the distribution as well as morphological and behavioural characteristics of *T. brucei* populations in the lymph nodes and the lymphatic vasculature, remains relatively understudied. In 1980, Tanner et al. reported the enrichment of *T. brucei* in the lymph nodes of rats and proposed these as important sites for parasite replication and antigenic variation (Tanner et al., 1980). While these observations were performed in fixed tissues and at a single time point of infection, current technology allowed us to investigate parasites *in situ*, *in vivo* with high spatial and temporal resolution. In the present work we used intravital microscopy to investigate *T. brucei* in 20 sets of lymph nodes, and in the lymphatic vasculature of the whole mouse body. We asked (a) whether parasite density varies, temporally and anatomically, throughout 20 days of infection across 20 lymph node sets and lymphatic vasculature studied in this work, and how it compares to blood vasculature parasite density; (b) whether the parasite population across lymph nodes differs to that in blood in terms of cell cycle, stumpy presence, morphology and behaviour; (c) whether the 20 sets of lymph nodes and lymphatic vasculature are remodeled during infection and what the consequences are for systemic homeostasis and (d) if exogenously modulating the vascular endothelium results in alterations to host-parasite interactions and survival.

## RESULTS

### Parasite density in lymph nodes is different from blood, and varies across lymph node groups

**Fig. 1.**
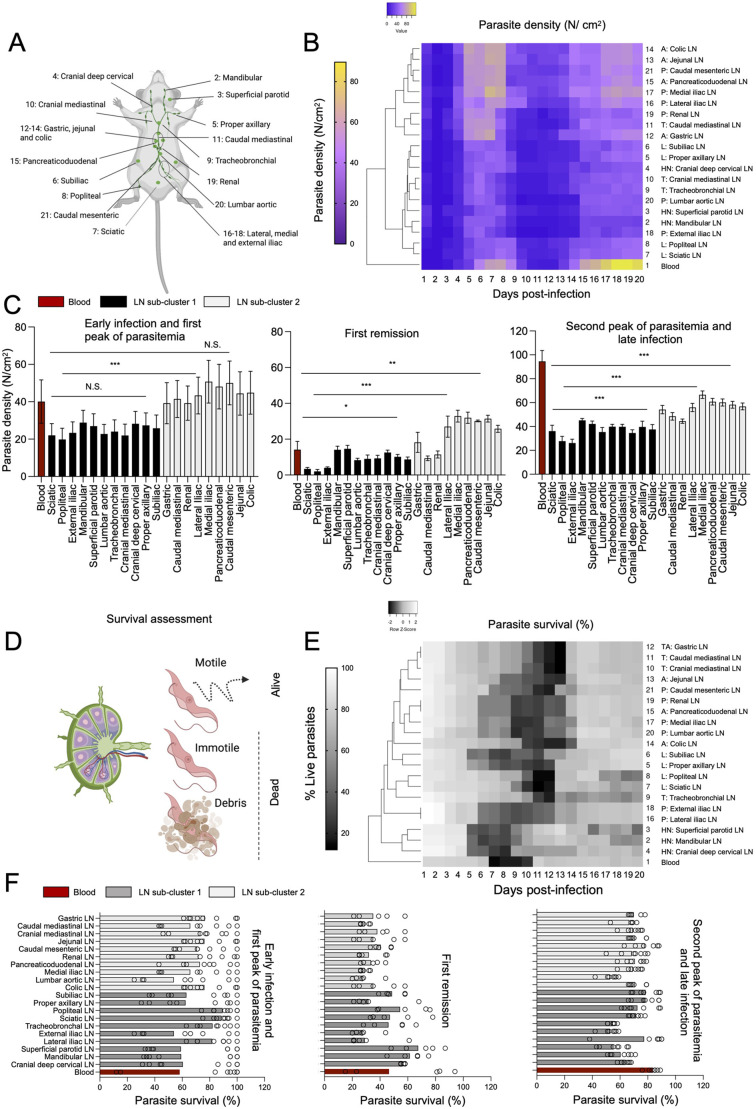
***T. brucei* density and survival across 20 lymph nodes and blood**. (A) Schematic representation of the anatomical localization of 20 lymph nodes distributed in the head and neck (HN, 2-4): mandibular, superficial parotid and cranial deep cervical; forelimbs and hindlimbs (grouped together as limbs (L, 5-8): proper axillary, subiliac, sciatic and popliteal; intra-thoracic (T: 9-11): tracheobronchal, cranial mediastinal and caudal mediastinal; intra-abdominal (A, 12-15): gastric, jejunal, colic and pancreaticoduodenal; and pelvic (P, 16-21): renal, lumbar aortic, lateral iliac, medial iliac, external iliac, and caudal mesenteric. For most subsequent figures, number 1 is given to blood (not marked in the schematic). (B,C) Parasite density in 20 lymph nodes and blood per day. Parasite density was calculated by measuring the extravascular area in at least 50 separate fields of view in each lymph node, and quantifying the parasite number in this area. Quantities are expressed as parasite number (N) per cm^2^. Dendrogram shows the blood as a separate element, and two sub-clusters, one containing LNs 2-10, 18 and 20, and one containing LNs 11-17, 19 and 21. (C) The average parasite density at three stages of infection [i.e. early infection and first peak of parasitemia (days 1-8); first remission (days 9-13); and second peak of parasitemia and late infection (days 14-20)] are shown for blood (red) and both lymph node sub-clusters (black bars and grey bars, respectively). One-way ANOVA tests were performed to determine significant difference between clusters. Significance is shown as N.S. (non-significant), *P*≤0.05 (*), *P*≤0.01 (**) and *P*≤0.001 (***). (D) Graphical representation of features used to score survival in lymph nodes and blood: motility (whether productive motility leading to parasite displacement or not) was considered a main feature for the classification as live parasite. Completely immotile parasites (including lack of any indication of flagellar beating) that included signs of cell death (including loss of fluorescence and/or debris) were classified as dead. (E) Parasite survival in 20 lymph nodes and blood. Parasite survival was calculated considering features mentioned in D. Quantities are expressed as parasite percentage calculated in at least 100 fields of view. Dendrogram shows the blood as a separate element, with no clear LN sub-clusters. (F) The average parasite survival at three stages of infection [i.e. early infection and first peak of parasitemia (days 1-8); first remission (days 9-13); and second peak of parasitemia and late infection (days 14-20)] are shown for blood (red) and two lymph node sub-clusters defined for (A-C) (dark grey and light grey bars, respectively). Circles represent the average parasite survival at each day post-infection.

In this work, we began by investigating *T. brucei* population density (expressed as number of parasites per cm^2^ of tissue) within 20 lymph node sets distributed in five groups: the head and neck (HN), forelimbs and hindlimbs (L), intra-thoracic (T), intra-abdominal (A), and pelvis (P) of C57BL/6J infected mice. The lymph nodes we studied include the mandibular, superficial parotid, cranial deep cervical, proper axillary, subiliac, sciatic, popliteal, tracheobronchial, gastric, jejunal, colic, pancreaticoduodenal, lateral iliac, medial iliac, external iliac, renal, lumbar, caudal mediastinal, and caudal mesenteric lymph nodes (schematic is shown in Fig. 1A). We infected 60 C57BL/6 mice with 3×10^4^ parasites and used three mice per day for analysis of all their lymph nodes, i.e. a total of either three or six lymph nodes (if they exist in pairs) were analysed per day, and a minimum of 50 fields of view per lymph node was used for quantification. We measured *T. brucei* density in these 20 lymph node sets over 20 days of infection, and compared it to the parasite density found in the blood vasculature of the liver. Data shown in Figs 1A,B,C and E are the average of 50 fields of view in three lymph node sets corresponding to three mice (See Fig. S1). Using hierarchical clustering, we observed that the blood is clustered separately from any of the lymph node sets (Fig. 1B). We then observed two large sub-clusters of lymph nodes: one which includes all lymph nodes of the head and neck, all lymph nodes of the limbs, two lymph nodes of the thorax (tracheobronchial and cranial mediastinal), and two lymph nodes of the pelvis (lumbar aortic and external iliac). The other large cluster includes all other pelvic and thoracic lymph nodes as well as all abdominal lymph nodes. All statistical analyses of comparisons to blood, within and between groups of lymph nodes are included in Table S1.

Based on the three sub-clusters described above (blood and lymph node clusters 1 and 2), we went on to investigate relative parasite densities over time across lymph nodes (Fig. 1C; Fig. S2, Table S2). We explored parasite density in three time sets: early infection and first peak of parasitemia (days 1-8, left panel), first remission (days 9-13, middle panel) and second peak of parasitemia and late infection (days 14-20, right panel) (Fig. 1C). On average, parasite burden during the early stage and first peak is 39.6, 24.6 and 44.6 parasites/cm^2^ in the blood and lymph nodes of cluster 1 and 2 respectively. The difference in parasite density between lymph node sub-clusters 1 and 2 at this stage, is statistically significant (*P*<0.01), but not the difference between either lymph node sub-cluster and blood (*P*>0.05) (Fig. 1C, left panel; Table S2). Analysis at higher temporal resolution showed that during this early stage, all lymph nodes are colonised faster than blood, and 24 h after infection they have, on average 8.2-fold (cluster 1) and 25.5-fold (cluster 2) higher parasite densities than blood (Fig. S2A; Table S2). Despite this faster initial colonization, parasite density in the blood rises quickly, reaching an average of 15 parasites/cm^2^ by day 3 post-infection, while lymph nodes in cluster 1 and 2 reach an average of 12.7 parasites/cm^2^ and 34 parasites/cm^2^, respectively. This represents a 192-fold increase relative to day 1 in the blood, a 30.3-fold increase in lymph nodes of cluster 1, and a 25.4-fold increase in lymph nodes of cluster 2 (Fig. S2B, Table S2). Parasite density continues to rise until its first peak at days 7-8 post-infection in the blood as well as lymph nodes of both clusters (Fig. 1B; Table S1).

This initial phase is followed by remission, where the minimum parasite density (second to parasitemia at day 1) across all 20 days is reached in both blood, and lymph nodes of both clusters. The average parasite density during this stage is 14.5, 8.7 and 24.3 parasites/cm^2^ in the blood and lymph nodes of clusters 1 and 2, respectively. Differences between both lymph node sub-clusters and the blood, and between both lymph node sub-clusters are statistically significant (*P*<0.01) (Fig. 1C, middle panel; Table S2). Finally, in the second peak of parasitemia, parasite density increases again in the blood and lymph node clusters 1 and 2 to 95, 36.7, and 56.2 parasites/cm^2^, respectively. Differences between both lymph node sub-clusters and the blood, and between both lymph node sub-clusters are statistically significant (*P*<0.001) (Fig. 1C, right panel; Table S2). Although the cumulative average is highest during the second peak of parasitemia in lymph nodes and blood, the absolute maximum parasite density across all days is reached in this third stage in the blood, but not in most lymph nodes (Table S2, Fig. S2C), as most lymph nodes show the maximum parasite density around days 5 and 7 post-infection, yet the difference between the maximum density at the first and second peaks of parasitemia is non-significant. Altogether, the early stage of infection and the remission phase seem to favour lymph node invasion over blood, and throughout all three infection stages parasite density variations in the lymph nodes remain minimal (Fig. S2D). The most parasite-enriched lymph nodes are proximal to the largest parasite reservoirs (e.g. pancreas and AT), and concentrate in the abdominal and pelvic regions of the mouse body.

Additional to total and relative parasite density, parasite survival in the lymph nodes varied greatly throughout infection. We scored viability using two criteria: motility and cell integrity. Loss of cell integrity included cell damage or signs of apoptosis, as well as debris (Fig. 1D). Complete loss of motility (without both, displacement and flagellar beating) is also indicative of loss of viability, and in previous work, we had shown that this varies across organs and time points of infection (De Niz et al., 2021). Intravital imaging suggests that dead parasites remain in intra- and extra-vascular locations for some time before being cleared by the immune system. Thus, it was important to evaluate viability early in our work, as a factor for inclusion/exclusion in the rest of the study. Hierarchical clustering showed that the blood is a separate cluster from the lymph nodes (Fig. 1E). The methodological design is shown in Fig. S3. Although large separate clusters of lymph nodes were not identified, a tendency towards lymph nodes within each anatomical group (i.e. HN, L, T, A, P) to show similar characteristics remained, and the grouping used for parasite density analysis was kept. Across all anatomical locations, parasite viability was maximal during early infection, and minimal around the first peak of parasitemia (prior to remission). Geometric means and individual points corresponding to each day are shown for three infection stages: early infection and first peak of parasitemia, remission, second peak of parasitemia and late infection. During early infection and first peak of parasitemia, the average parasite survival was 58.2, 67.8 and 73.1% in the blood and lymph node sub-clusters 1 and 2, respectively (Fig. 1F left panel, Table S3). During remission this fell to 46.5, 46.0 and 33.1% in the blood and lymph node sub-clusters 1 and 2, respectively (Fig. 1F middle panel; Table S3). This was followed by a rise in survival during the second peak of parasitemia and late infection, reaching 83.6, 64.2 and 71.6% survival in the blood and lymph node sub-clusters 1 and 2, respectively (Fig. 1F right panel; Table S3). Although on average survival was highest in the blood, an absolute minimum of 12% survival was recorded in the blood, while the minimum survival in lymph node sub-clusters 1 and 2 was of 29.2 and 24.4%, respectively (Fig. S2E). Therefore, survival fluctuations were highest in the blood (as explored in Table S3 and Fig. S2F). To avoid mortality-related confounding factors, only live parasites were considered for all further analysis. Altogether, based on these observations, we hypothesise that *T. brucei* successfully adapt to lymph nodes, and display high viability in these organs during infection.

### The mouse lymph nodes harbour a rapidly replicating parasite population, and are a less favourable environment for stumpy presence/formation

**Fig. 2.**
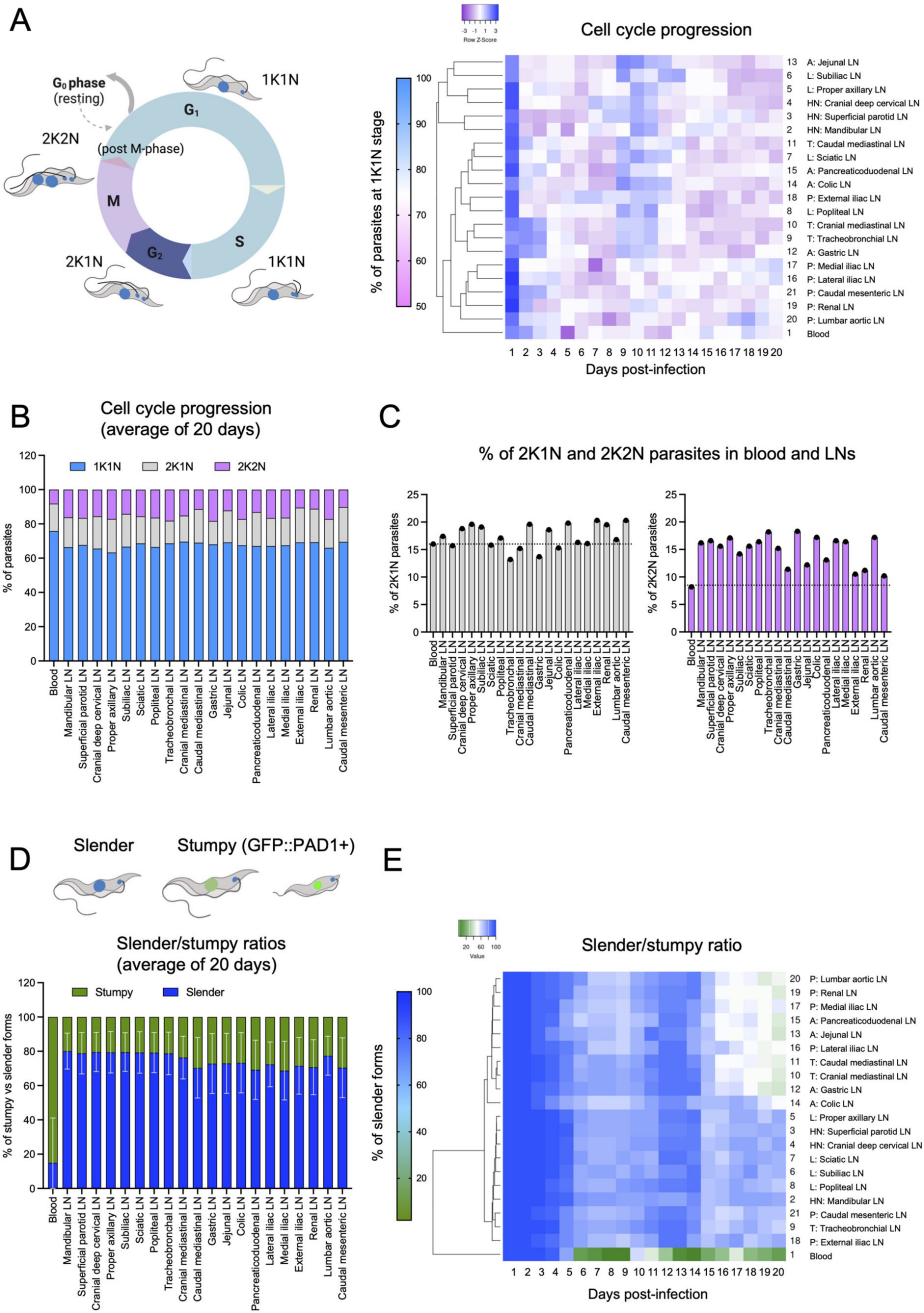
***T. brucei* cell cycle progression and stumpy/slender presence across 20 lymph nodes and blood**. (A) Schematic representation of *T. brucei* cell cycle, showing 1 kinetoplast and 1 nucleus (1K1N) in the G and S phase (shown in blue), 2K2N in the G2 phase (shown in dark blue), and 2K2N in M and the post-M phase (shown in purple). Parasite 1K1N, 2K1N and 2K2N percentages in 20 lymph nodes per mouse in three mice per day, and blood were calculated in at least 50 separate fields of view. Dendrogram shows only the 1K1N population, and shows the blood as a separate element, with sub-clusters corresponding, to some extent, to anatomical location (i.e. HN, L, T, A, P). (B,C) Relative percentages of each sub-population are shown for all organs measured. (C) shows the separate percentages of the 2K1N and 2K2N populations. The dotted line shows the blood average, which is not significantly different for the 2K1N population, but significantly different for the 2K2N population, as determined by Student's *t*-test for each LN relative to blood, and one-way ANOVA for LN groups relative to blood and to each other. (D) Schematic representation of slender and stumpy *T. brucei* populations. For the purpose of this work, GFP-negative forms were defined as slender forms, while GFP-positive forms were defined as stumpy. Importantly, GFP-positive forms did not only include fully differentiated stumpy forms, however this was mostly relevant to the blood, as the majority of parasites in the LNs were GFP-negative. Slender (blue) and stumpy (green) percentages in 20 lymph nodes and blood were calculated in at least 50 separate fields of view per lymph node per mouse. Graph shows the geometric mean of all 20 days of infection. Bars represent the geometric SD. (E) Dendrogram for the slender/stumpy ratio over 20 days of infection shows the blood as a separate element, with two main sub-clusters, one including all HN and L lymph nodes, and one including all A and most P and T lymph nodes.

Having detected the different parasite densities and survival patterns of *T. brucei* across the 20 lymph node sets, we went on to ask whether the parasite population within the lymph nodes differs significantly from the bloodstream population and between lymph node sets. We began by analysing cell-cycle progression among the parasites populating each lymph node set, by nucleus and kinetoplast quantification (Fig. 2A left panel). Methodological details corresponding to this set of experiments are shown in Fig. S4. Hierarchical clustering showed two major clusters, one of which includes the blood, all pelvic (P), all thoracic (T) and most abdominal (A) lymph nodes. The other cluster includes all head and neck (HN) lymph nodes. Limb (L) lymph nodes are distributed across both major clusters (Fig. 2A; Table S4). We found that, on average, all lymph nodes have significantly lower percentages of parasites at 1K1N stage than the average parasite population in blood (*P*<0.01) (Fig. 2B; Table S4). While the average percentage of parasites at 1K1N stage in the blood was 73.2%, it was 66.6, 66.2, 69.1, 67.9 and 68.1% in HN, L, T, A and P lymph node sets, respectively. This difference between lymph nodes and blood was significant in all cases (Table S4). However, the difference within lymph node groups in HN, L, T, A and P was not significant. The average percentage of parasites at 2K1N stage in the blood was 18.6%, while it was 17.3, 17.9, 16, 16.9 and 18.3% in HN, L, T, A and P lymph node sets, respectively. The difference between lymph nodes and blood was mostly not significant, while within most groups (all except L) the differences were significant (Fig. 2C left panel; Table S4). Finally, the average percentage of parasites at 2K2N stage in the blood was 8.4%, while it was 16.1, 15.8, 14.9, 15.2 and 13.7% in HN, L, T, A and P lymph node sets, respectively. This difference was significant for all lymph nodes compared to blood (*P*<0.001), but not significant within lymph node groups, except for A lymph nodes (Fig. 2C right panel; Table S4).

Given the significant difference in 1K1N and 2K2N forms in the blood compared to all lymph nodes, we hypothesised that differences might exist in the presence of stumpy and slender forms in the blood and lymph nodes. We went on to score the presence of stumpy and slender forms by the use of a PAD1:GFP reporter parasite line (Fig. 2D-E). Methodological details are shown in Fig. S5. Upon close analysis of the parasite population in these organs, we observed that expression of GFP was not restricted to morphological stumpy forms. Rather, GFP was expressed in a range of intensities and across a range of parasite morphologies, consistent with the growing interest in ‘intermediate forms’. We classified parasites into nine categories based on morphology and GFP expression as described in Fig. S6. We found that all parasite forms are present across blood and all lymph nodes. However, when morphological stumpy forms are excluded from the analysis, the overall blood population shows enrichment of slender forms and long and wide parasites expressing GFP. The lymph nodes on the other hand, show a significant enrichment of long and/or wide parasites that do not express GFP (Fig. S6A-C). An immediate question we wanted to answer was whether cell cycle progression was different among these so-called intermediate forms. We found that despite showing identical morphological features, parasites expressing PAD1-GFP showed little to no signs of replication (i.e. were a 1K1N enriched population), while parasites not expressing PAD1-GFP were all enriched in 2K1N and 2K2N parasite forms (Fig. S6D). While this observation raises interesting future questions regarding the function, characteristics, and significance of ‘intermediate forms’, we decided to focus, for this section, on the presence or absence of PAD1-GFP. Hierarchical clustering (Fig. 2E) shows the blood as a separate cluster, and two main lymph node sub-clusters, one including all HN and L lymph nodes, and another including most of the T, A and P lymph nodes. We calculated the geometric mean of the percentage of slender forms in blood to be 15.01%, while it was 79.56 and 79.41% in lymph node cluster 1 (HN and L), and 75.1, 72.1, and 71.9% in lymph node cluster 2 (T,A and P) (Fig. 2D-2E; Table S5). Temporally resolved quantifications showed significantly different patterns of slender/stumpy presence throughout infection. In the blood, there is a steep decrease in slender forms by the first peak of parasitemia (days 6-9), reaching the absolute minimum (i.e. stumpy maximum %) at days 8-9. During the first half of the infection, the average slender percentage is 54.6%, while in the second half it is 21.3% reaching a minimum of 2% (Fig. S7A, Table S5). In lymph node cluster 1 (HN and L), slender presence remains significantly higher throughout infection, with an average of 85.7% during the first 10 days, 74.9% during the last 10 days, and a minimum of 63% at any point during the infection (which occurs between days 15-20). In lymph node cluster 2 (T, A and P), slender presence remains significantly higher than in blood throughout infection, with an average of 81.3% during the first 10 days, 67.4% during the last 10 days, and a minimum of 40% at any point during the infection (between days 16 and 20) (Fig. S7B, Table S5). Together, these observations suggest that the lymph nodes are an important reservoir that favours parasite replication, which coincides with previous reports of higher *T. brucei* proliferation rates in rat lymph nodes (Tanner et al., 1980). Moreover, it suggests that the lymph node environment is less favourable for stumpy formation/presence than blood. This finding has key implications for our understanding of the role of extravascular reservoirs (the lymphatic system in particular) for *T. brucei* transmission.

### The mouse lymph nodes harbour a parasite population morphologically different from the one in blood

**Fig. 3.**
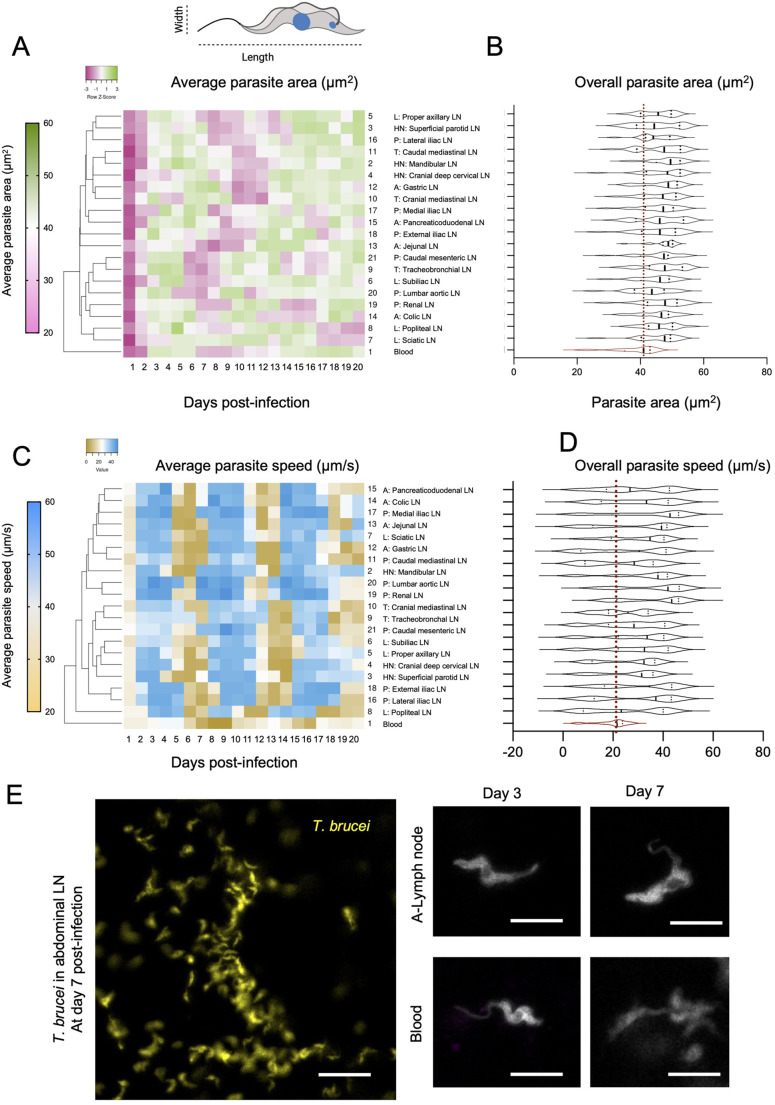
***T. brucei* morphology and behaviour across 20 lymph nodes and blood**. (A) Schematic representation of *T. brucei* length and width as used to calculate parasite area. Parasite length and width were calculated in at least 50 fields of view in 20 lymph nodes and blood per mouse in a total of three mice per day. Dendrogram shows the average parasite area at each day of infection. It shows the blood as a separate element, with no clear sub-cluster of lymph nodes. (B) Violin plots showing the distribution of parasite areas for all organs analysed, during 20 days of infection. Blood is shown in red. (C) Parasite speed was calculated in at least 50 fields of view in 20 lymph nodes and blood per mouse. Dendrogram for the average parasite speed at each day of infection shows the blood as a separate element, with no major lymph node sub-clusters yet some degree of grouping by anatomical location (i.e. HN, L, T, A, P). (D) Violin plots showing the distribution of parasite speed for all organs analysed, during 20 days of infection. Blood is shown in red. (E) Representative images of parasite morphology in blood and LNs. Scale bars: 20 µm.

In addition to replication rate and stumpy/slender differences, we observed that parasites in the lymph nodes display an altered morphology compared to blood-circulating forms. Namely, in all lymph nodes analysed, parasites become progressively longer and wider than parasites conventionally found in blood (Fig. 3A,B; Figs S8 and S9). This is summarised in Tables S6-8 and coincides with previous findings reported in the lymph nodes of infected rats (Tanner et al., 1980). Note: for all cases, parasite measurements were done in morphological slender forms to avoid confounders due to stumpy morphology. While we had done a semi-quantitative classification for Fig. S6, here we went on to consider a fully quantitative approach for parasite analysis. Methodological details are shown in Fig. S8. Morphological changes were a continuous rather than a spontaneous event, with the parasite population displaying variable morphology throughout infection. As a control to a potential confounder due to cell cycle stage, we began by comparing the length and width of the parasite population across cell-cycle stages (i.e. 1K1N, 2K1N, 2K2N) and we found that although differences exist in length and width between cell-cycle stages, in all cases, differences between blood and LN population was significant for each cell cycle sub-group (Fig. S9A) (*P*<0.01). Hierarchical clustering for width measurements showed the blood as a separate cluster to all lymph nodes, with no other significant clusters between the lymph node sets (Fig. S9B-S9C). In the blood, the average parasite width throughout 20 days of infection was 2.13 µm. Width varied between a minimum (min) of 1.4 µm at day 1 post-infection, and a maximum (max) of 2.5 µm at days 18-19 post-infection. This represents a 78% increment in width. In the lymph nodes, the variation recorded was on average 46.6%, and ranged between 18.7% (Jejunal LN) and 75.9% (Cranial deep cervical LN). The average width in the blood parasite population (2.13 µm) was significantly lower than the width of parasites in all lymph nodes (Table S6), which was on average, 2.5 µm. In the lymph node groups previously defined by anatomical location (HN, L, T, A, P), the average parasite width was 2.51 µm (min: 1.88 µm, max 2.82 µm) in the HN group; 2.48 µm (min: 1.91 µm, max: 2.76 µm) in the L group; 2.47 µm (min: 1.99 µm, max: 2.77 µm) in the T group, 2.46 µm (min: 2.06 µm, max: 2.74 µm) in the A group and 2.45 µm (min: 1.80 µm, max: 2.8 µm) in the P group (Fig. S9C). In all groups, changes in width were time-dependent, with the highest widths recorded at the peaks of parasitemia, and the smallest widths recorded either at the beginning of infection or during the remission phase (Table S6). Within-group differences were non-significant for L, T, A and P groups (*P*>0.05), but significant for the HN group (*P*=0.04). Considering all lymph nodes across all time points, the differences in width were not significant (*P*=0.1).

Similar to width, variations in parasite length also occurred throughout infection. Hierarchical clustering for length measurements showed the blood as a separate cluster to all lymph nodes, with no other significant clusters between the lymph node sets (Fig. S9D-E). In the blood, the average parasite length throughout 20 days of infection was 17.7 µm. Length varied between a minimum of 15.76 µm at day 1 post-infection, and a maximum of 18.66 µm at day 17 post-infection (Table S7). This represents an 18.4% increment in length. In the lymph nodes, the variation recorded was on average 28.2%, and ranged between 21.5% (Cranial deep cervical LN) and 38.2% (Jejunal LN). The average length in the blood parasite population (17.7 µm) was significantly lower than the length of parasites in some lymph nodes [all in HN, popliteal (L), cranial mediastinal (T), gastric (A), jejunal (A), pancreaticoduodenal (A), external iliac (P), and caudal mesenteric (P) (Table S7), but not others]. The average parasite length in all lymph nodes was 18.4 µm. In the lymph node groups previously defined by anatomical location (HN, L, T, A, P), the average parasite length was 18.58 µm (min: 16.07 µm, max: 20.02 µm) in the HN group; 18.18 µm (min: 15.75 µm, max: 20.02 µm) in the L group; 18.27 µm (min: 15.69 µm, max: 20.25 µm) in the T group; 18.83 µm (min: 15.9 µm, max: 20.68 µm) in the A group, and 18.25 µm (min: 15.49 µm, max: 19.96 µm) in the P group (Fig. S9E). In all groups, changes in length were time-dependent, with the highest lengths recorded at variable times, but the smallest lengths recorded either at the beginning of infection or during the remission phase. Within-group differences were non-significant for HN, T and P groups (*P*>0.05), but significant for the L and A groups (*P*=0.02 and 0.006, respectively). Considering all lymph nodes and all time points, the differences in length were significant (*P*=0.01). While the average length was not significantly different between lymph nodes and blood in all cases, the maximum length reached by parasites in any lymph node (21.38 µm), was significantly different to the maximum reached in blood (18.66 µm). Equally, the difference between minimum and maximum lengths was highest in the lymph nodes (between 21.4 and 38.2% increase in length) compared to blood (18.4%) (Table S7).

Considering length and width parameters, we went on to determine whether the parasite area (defined as a proxy) was similar in blood and lymph nodes (Fig. 3A,B; Table S8). Hierarchical clustering for area measurements showed the blood as a separate cluster to all lymph nodes, with no other significant clusters between the lymph node sets (Fig. 3A). In the blood, the average parasite area throughout 20 days of infection was 37.5 µm^2^. Parasite area ranged between a minimum of 21.9 µm^2^ at day 1 post-infection, and a maximum of 45.49 µm^2^ at day 18 post-infection. This represents a doubling in area (>100% increment). In the lymph nodes, the area variation recorded was on average 74.9%, and ranged between 47.6% (Proper axillary LN) and 109.2% (Cranial Deep Cervical LN). The average area in the blood parasite population (37.54 µm^2^) was significantly lower than the area of parasites in all lymph nodes (Table S8). The average parasite area in all lymph nodes was 45.9 µm^2^. In the lymph node groups previously defined by anatomical location (HN, L, T, A, P), the average parasite area was 46.71 µm^2^ (min: 31.66 µm^2^, max: 55.29 µm^2^) in the HN group; 45.19 µm^2^ (min: 31.51 µm^2^, max: 53.2 µm^2^) in the L group; 46.16 µm^2^ (min: 31.99 µm^2^, max: 54.35 µm^2^) in the T group, 46.46 µm^2^ (min: 34.07 µm^2^, max: 53.82 µm^2^) in the A group, and 44.79 µm^2^ (min: 28.37 µm^2^, max: 53.52 µm^2^) in the P group (Fig. 3B, Table S8). Consistent with our separate findings for width and length, in all groups, changes in area were time-dependent, with the highest areas recorded during parasitemia peaks, and the smallest areas recorded mostly at the beginning of infection. Within-group differences were non-significant between lymph node groups (*P*>0.05). Considering all lymph nodes at all time points, the differences in parasite area were non-significant (*P*=0.13). The maximum area reached by parasites in any lymph node (51.6 µm^2^ to 56.5 µm^2^), was significantly different to the one reached in blood (45.49 µm^2^) (Table S8). Altogether, we conclude that the parasite population in the lymph nodes is significantly different to the one found in blood, with parasites in lymph nodes having larger areas than those in blood.

### The mouse lymph nodes harbour a fast-moving parasite population, behaviourally different to the one in blood

In addition to morphological changes, we went on to measure speed of motion (displacement as a function of time) in blood and lymph nodes (Fig. 3C,D; Table S9). Methodological details are shown in Fig. S10. Notably, in order to eliminate possible confounders related to different lymph flow, we ligated the lymphatic vasculature to abrogate host-induced movement *in vivo*. Hierarchical clustering for speed showed the blood as a separate cluster to all lymph nodes, without clear major sub-clusters arising for the LNs (Fig. 3C). The geometric mean of parasite speed in blood throughout 20 days of infection was 14.74 µm/s. Parasite speed ranged between a minimum of 2.13 µm/s at the beginning of the remission phase, and a maximum of 27.46 µm/s at day 4 post-infection. This represents a 27-fold change in speed range. In the lymph nodes, parasites showed drastic changes in speed but the minimum speed was significantly higher than in blood. Considering all lymph nodes, the average speed was of 24.78 µm/s. The average difference between the minimum and maximum registered speed was a 7.26-fold change, with the highest range registered for the HN lymph nodes (10.56-fold change), and the lowest for T lymph nodes (5.15-fold change). The average parasite speed in the blood population (17.69 µm/s) was significantly lower than the speed of parasites in all lymph nodes (Table S9). In the lymph node groups previously defined by anatomical location (HN, L, T, A, P), the geometric mean of parasite speed was 24.76 µm/s (min: 4.07 µm/s, max: 42.95 µm/s) in the HN group; 23.36 µm/s (min: 6.89 µm/s, max: 42.13 µm/s) in the L group; 27.39 µm/s (min: 10.67 µm/s, max: 46.61 µm/s) in the T group; 21.09 µm/s (min: 5.91 µm/s, max: 41.24 µm/s) in the A group, and 26.9 µm/s (min: 7.29 µm/s, max: 47.3 µm/s) in the P group (Fig. 3D). Consistent with our findings for differences in morphology, in all groups, changes in speed were infection time-dependent with drastic variations occurring within a short period of time during both parasitemia waves (Fig. S11A-D, Table S9). Within-group differences were non-significant between most lymph node groups (*P*>0.05) except the T lymph nodes (*P*=0.02). Considering all lymph nodes an all time points, the differences in parasite speed were significant (*P*=0.04). Altogether, the maximum speed reached by parasites in any lymph node (50.92 µm/s), was significantly different to the one reached in blood (27.46 µm/s) (Fig. S11C,D; Table S9). Representative images of parasites in blood and lymph nodes are shown in Fig. 3E. Altogether, our data shows that in addition to morphological differences, the parasite population is behaviourally different to the one found in blood, with parasites displaying faster swimming in lymph nodes.

**Fig. 4.**
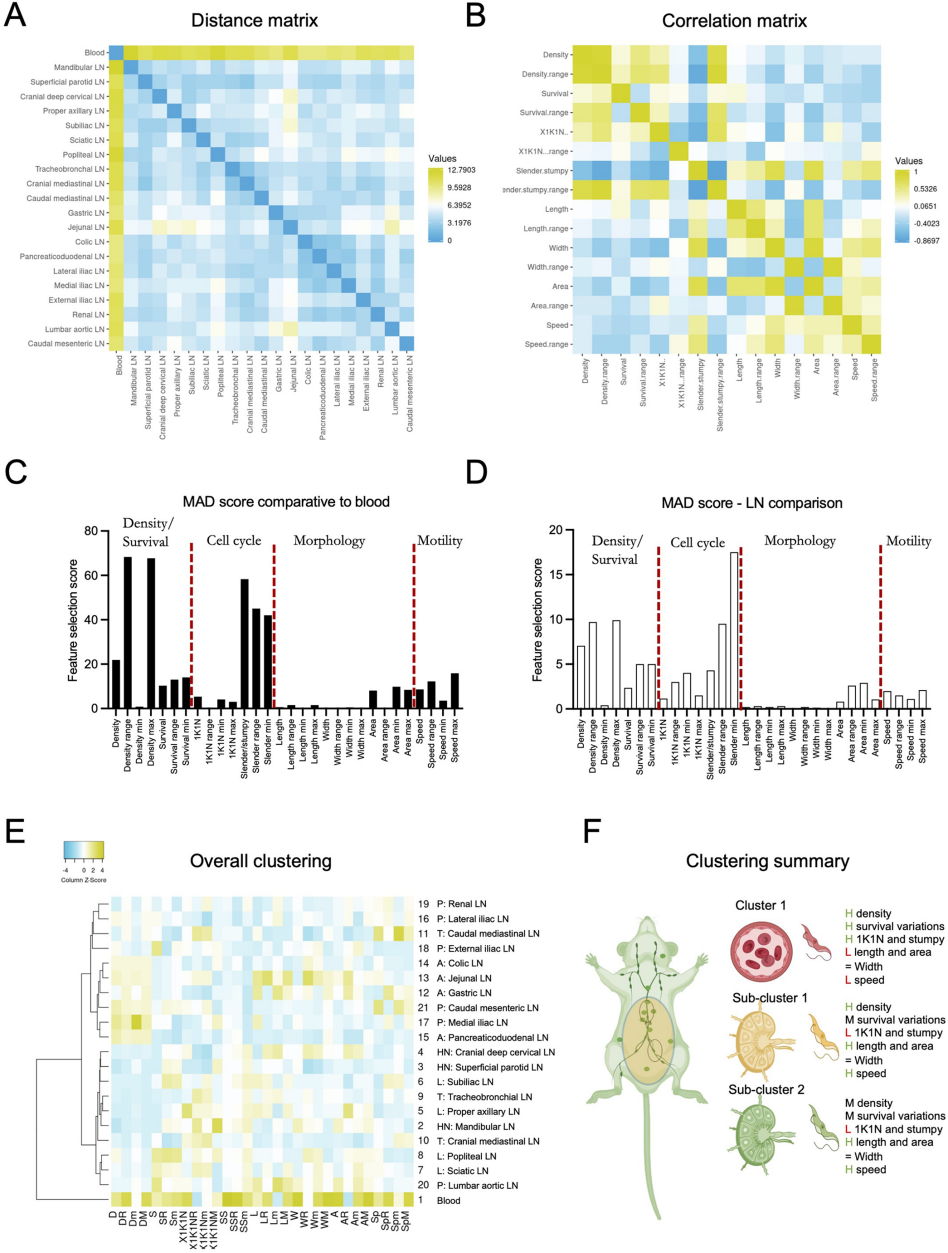
***T. brucei* blood population is significantly different to LN population, with the most defining selection factors being parasite density and slender/stumpy ratios**. Considering parasite density, survival, cell cycle progression, slender/stumpy ratios, length, width, area and speed, including average, range, minimum and maximum values, various factor selection features were calculated. (A) Distance matrix based on all aforementioned factors shows that the largest separation (12.8) exists for blood relative to any lymph node, while the distance between any lymph node is lower than 6. (B) A correlation matrix shows high positive correlation (>0.5) between density, survival, and cell cycle progression, as well as between length, width, area and speed. (C) MAD was used for factor selection calculation between blood and LNs. The highest score was reached by parasite density range and maximum, followed by slender/stumpy average, range and minimum. (D) MAD was used for factor selection calculation between LNs. The highest score was reached by slender/stumpy minimum and range, followed by density range and density maximum. (E) Considering all aforementioned features we investigated whether lymph node clustering existed. We confirmed the existence of three sub-clusters: one defined by the blood, one defined by all HN and L lymph nodes (as well as a P and 2* T* lymph nodes), and one defined by all A and remaining P LNs). (F) Schematic representation of overall findings regarding parasite populations in the LNs and blood.

Finally, we considered all previously explored criteria (i.e. parasite density, parasite survival, cell cycle progression, stumpy/slender proportions, length, width, area, and speed) to perform a global analysis of differences and similarities between parasites in lymph nodes and blood, and between different lymph node sets (Fig. 4). Fig. 4A shows a distance matrix, which allowed us to explore whether altogether (a) the lymph node parasite population was different to the one in the blood, and (b) whether any sub-clustering existed within the 20 lymph node sets explored. The distance matrix showed the highest difference for blood (12.79). This is consistent with the majority of the analyses done for each independent criterion. We then performed feature correlation and selection methods to determine which of the features analysed were most relevant for predicting parasite location (i.e. between blood and lymph nodes, as well as within lymph node sets). A correlation matrix (Fig. 4B; Table S10) shows high correlation scores (>0.5) between parasite density, survival and cell-cycle progression, as well as between parasite length, width, area and speed. Slender % has high negative correlation scores (<-0.4) relative to density, survival and cell-cycle progression.

For feature selection, we performed a median absolute deviation (MAD) analysis, which showed that the most important features for distinguishing blood from lymph node *T. brucei* populations (Fig. 4C; Table S11), include those relative to parasite density [specifically the density range (difference between maximum and minimum) and the density maxima], with MAD scores of 68.3 and 67.7, respectively. These were followed by features related to slender/stumpy ratios including the average, the minimum slender percentage registered, and the range), with MAD scores of 58.3, 42 and 45, respectively. Amongst the remaining features, speed range and maxima had the highest MAD scores (12.3 and 15.9, respectively). Considering only inter-lymph node comparisons, the same features as those previously discussed (i.e. parasite density and slender/stumpy ratios) had the highest MAD scores, with the slender/stumpy minima having the highest score (17.5). Overall, MAD scores for inter-LN differentiation were on average 4-fold lower than those recorded for the blood/LN differentiation (Fig. 4D; Table S11).

While we previously investigated each feature independently to determine whether any clusters existed among LN sets and blood, we went on to investigate clustering based on a composite comparison. Hierarchical clustering considering all criteria (i.e. parasite density, parasite survival, cell-cycle progression, stumpy/slender proportions, length, width, area, and speed) confirmed the blood as a separate cluster, and showed two large lymph node sub-clusters, one including all HN and L as well as most T lymph nodes, and one including all A and all P lymph nodes (Fig. 4E,F). This is consistent with most findings recorded for individual criteria, and highlights an important difference between lymph node groups based on their anatomical location, and the organ reservoirs they neighbour.

### Infection-induced structural remodelling of the lymphatic system correlates with accumulation of parasitised free fluid

**Fig. 5.**
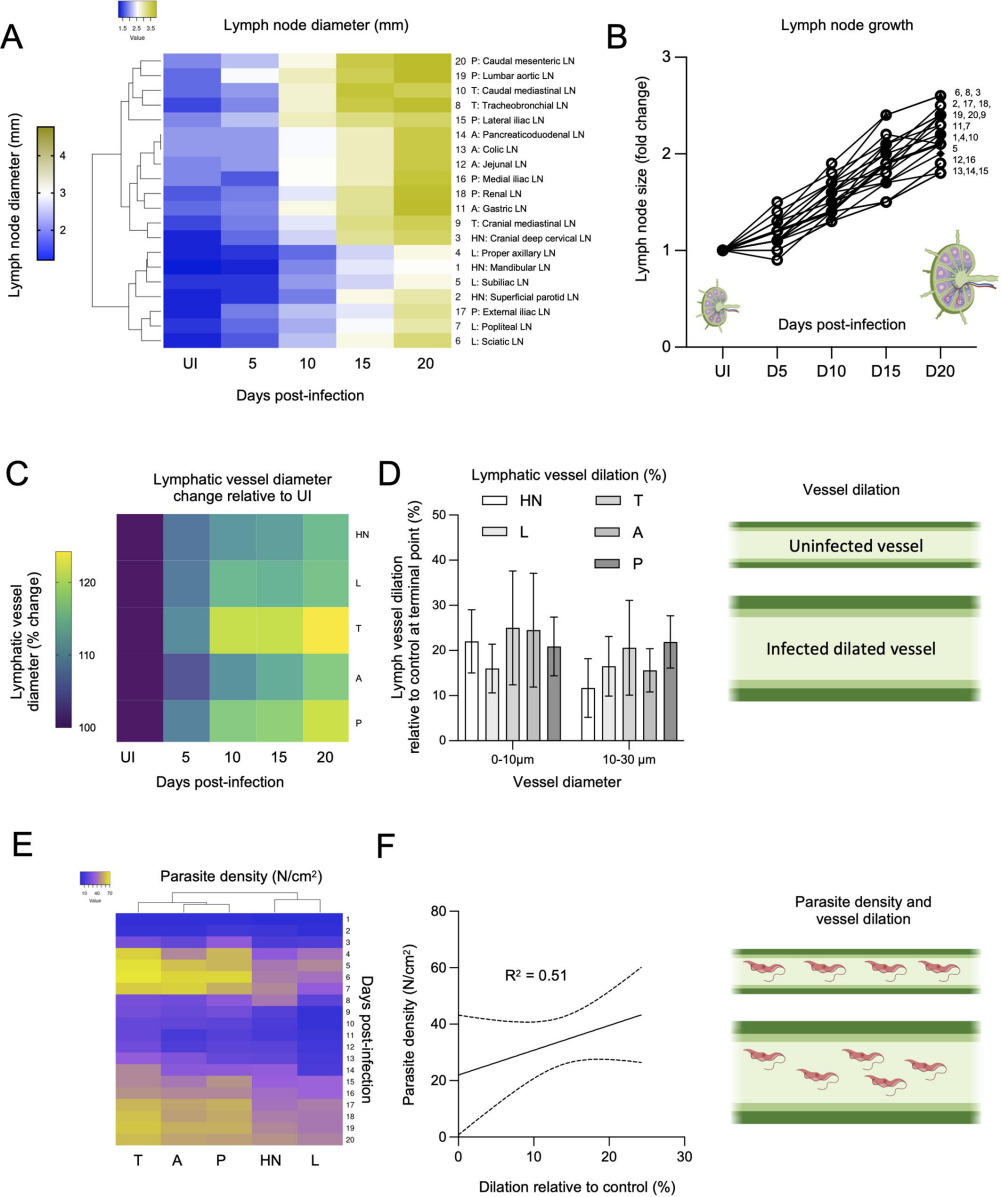
***T. brucei* infection results in significant lymphatic system remodelling and deleterious host pathology**. (A) Lymph nodes diameters were measured at four different times of infection, namely at days 5, 10, 15 and 20, as well as in uninfected mice. Hierarchical clustering showed three clusters with slightly different patterns of lymph node remodelling: most HN and L lymph nodes underwent significantly lower and more gradual enlargement than LNs in the T, A, and P areas. (B) Analysis on the fold change in size by day 20 relative to uninfected mice shows that most lymph nodes increase their size by between 1.8- and 2.6-fold. Moreover, LNs continue to increase in size throughout infection. (C) The lymphatic vasculature diameter was measured in one representative vasculature for each anatomical location (HN, L, T, A, P). An increase of between 15 and 24% in diameter was recorded for all representative lymphatic vessels, with the T and P lymphatic vasculature showing the greatest dilation. (D) Analysis of whether dilation occurred equally in vessels of different diameter. We observed a tendency towards smaller vessels (0-10 µm) to undergo greater dilation than larger vessels (10-30 µm), this difference was not statistically significant. Error bars shown represent s.d. (E) Parasite density in the lymphatic vasculature was calculated in HN, L, T, A, P locations, with T, A, P (the most highly dilated vasculature) showing highest parasite density. (F) Correlation between parasite density and vessel dilation showed an R^2^ score of 0.51.

In addition to our investigation on lymph node colonization and remodelling upon *T. brucei* infection, we also observed that the lymph nodes are significantly remodelled throughout infection (Fig. 5A,B; Table S12). Lymph node diameters were measured in uninfected mice, and at days 5, 10, 15 and 20 post-infection. Methodological details are included in Fig. S12. Hierarchical clustering identified three major sub-clusters (Fig. 5A). Although there was no clear sub-division coinciding fully with anatomical location, there was still a tendency for some lymph nodes to cluster together. One sub-cluster included most HN and all L lymph nodes, another included all A and most P lymph nodes, and one included most T and the remaining P lymph nodes. The average uninfected LN diameter was 1.6 mm, with HN, L and T lymph nodes being smaller (1.3, 1.4, 1.6 mm, respectively) than A and P lymph nodes (1.9, 1.8 mm, respectively). Most lymph nodes showed a significant increase in size by day 5 post-infection (*P*<0.001) to an average diameter of 1.8 mm; to 2.4 mm by day 10 post-infection (*P*<0.001); to 2.9 mm by day 15 post-infection (*P*<0.001), and to 3.4 mm by day 20 post-infection (*P*<0.001). This represented a 2.2-fold increase in size by day 20 post-infection, on average, with the greatest increase observed in T, A and P lymph nodes (Fig. 5B; Table S12).

We then went on to investigate whether the lymphatic vasculature also experienced anatomical changes throughout infection. Methodological details are included in Fig. S13. We focused on vessel dilation, and selected vasculature of various diameters per anatomical location, namely, the mandibular LN (HN), the popliteal LN (L) the tracheobronchial LN (T), the pancreaticoduodenal LN (A), and the external iliac LN (P). For lymphatic vessel identification, we injected mice with A647-conjugated LYVE-1 antibody. Irrespective of anatomical location, we identified a significant lymphatic vessel dilation by day 5 post-infection, with significant increases every 5 days of infection (Fig. 5C; Table S13). At the terminal point of infection (day 20), we identified that lymphatic vessels of medium diameter (0-10 µm) were enlarged by 22% (HN), 16% (L), 25% (T), 25% (A) and 21% (P) relative to uninfected mice. While larger lymphatic vessels (10-30 µm) also showed significant enlargement, it was not as prominent as the one found in smaller vessels. Larger lymphatic vessels showed a 11.7% (HN), 16.5% (L), 20.5% (T), 15.6% (A), and 21.9% (P). Altogether, vessels linked to the T, A and P lymph nodes showed the greatest modifications (Fig. 5D; Table S13). Although we explored whether vessel enlargement and parasite density (in the lymphatic vasculature) (Fig. 5E) were correlated, we found an overall R^2^ value of 0.51, suggesting weak correlation (Fig. 5F).

**Fig. 6.**
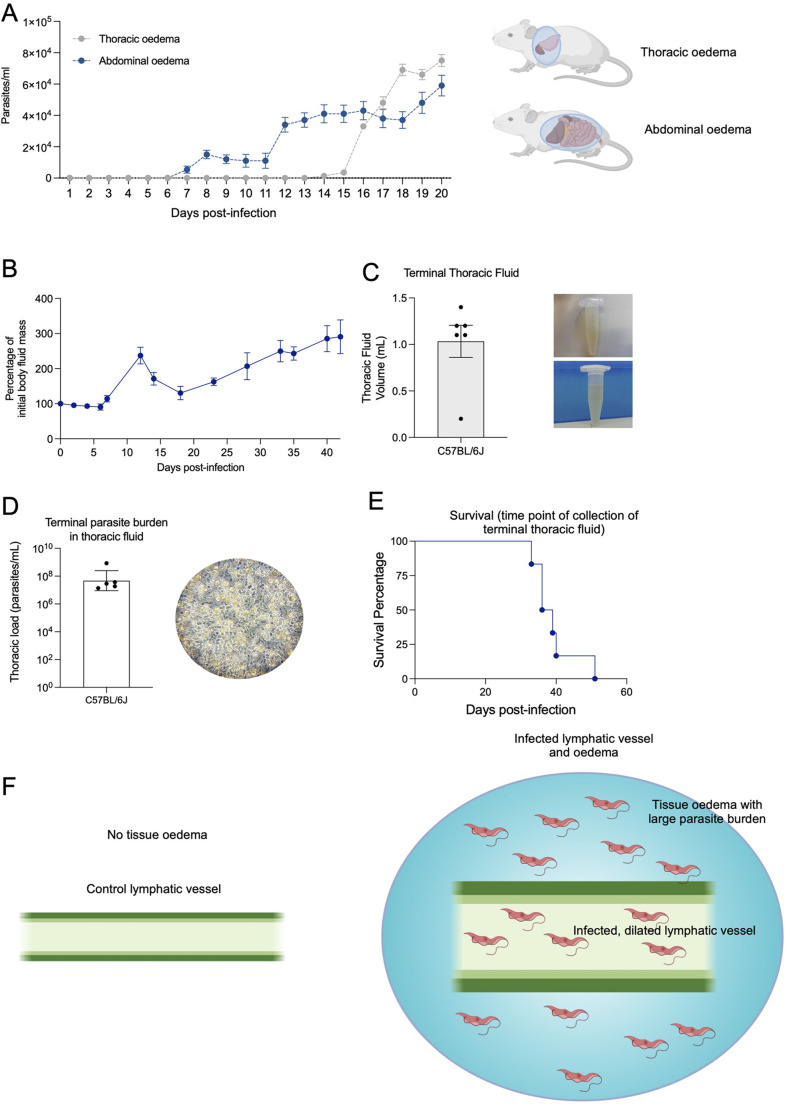
***T. brucei* infection results in significant oedema across the mouse body**. (A) Parasitemia (expressed as parasites/ml) was calculated in the free fluid found in the thorax (grey lines) and abdomen (blue lines) of mice at each point of intravital surgery. While parasitemia in the free abdominal fluid gradually increased from day 6, as infection progresses, parasitemia in the thorax showed a sharp increase from day 15 of infection. By day 20 post-infection, the free abdominal fluid had a parasitemia of 5.9×10^4^ parasites/ml, while the free thoracic fluid reached 7.5×10^4^ parasites/ml. Dots represent average parasitemia. Error bars shown represent s.d. (B) Total free fluid mass was measured by nuclear magnetic resonance throughout infection and then normalised to baseline. Free fluid mass increased by 2.9-fold by day 42 post-infection. (n=5 mice). (C) Parasite burden of thoracic fluid and (D) volume isolated from moribund mice (33-51 days post-infection, *n*=6 mice). (D) Survival curve of infected mice, showing the time of fluid collection.

Given this result suggesting lymph node and lymphatic vessel pathology, as well as our constant findings during intravital surgeries, of the presence of liquid consistent with oedema in the thoracic, and abdominal-pelvic cavities, we went on to analyse the isolated fluid from these compartments (Fig. 6A; Table S14). Methodological details are included in Fig. S14. Significant free fluid was detectable in the abdomen from day 7 post-infection (i.e. around the first peak of parasitemia), while it was only detectable in significant amounts in the thorax from day 14 post-infection. Upon analysis of the fluid for presence of parasites, we found 5.4×10^3^ parasites/ml and 1.4×10^3^ parasites/ml in the abdomen and thorax respectively. This increased 10-fold and 50-fold by day 20 post-infection in the abdomen and thorax, respectively. This is consistent with a gradual increase of parasites in the abdomen (1.3-fold daily), but a drastic increase in free fluid and parasite presence in the thorax (2.8-fold daily). Upon investigating free fluid consistent with lymphatic system pathology until day 42 post-infection, we observed fluctuations between −10% and +62% within the first 20 days of infection, relative to free fluid in uninfected mice. Methodological details are included in Figs S15 and S16. This drastically increased during the next 22 days culminating in +190% at day 42 post-infection relative to uninfected mice (Fig. 6B; Table S14). Towards the end of mouse survival (which varied among different mice, between days 33 and 51) a large amount of free fluid was extracted from the thorax of six moribund infected mice (i.e. between 0.2 and 1.4 ml), and this fluid was found to be heavily parasitised, with between 1.37×10^7^ and 8.55×10^8^ parasites/ml (Fig. 6C-E; Table S14). Together, these findings show that a *T. brucei* infection leads to significant structural remodelling of the lymphatic system, which correlates with the accumulation of potentially lethal amounts of parasitised oedema (Fig. 6F).

### Blocking the lymphatic vascular receptor LYVE-1 is detrimental for parasite survival

**Fig. 7.**
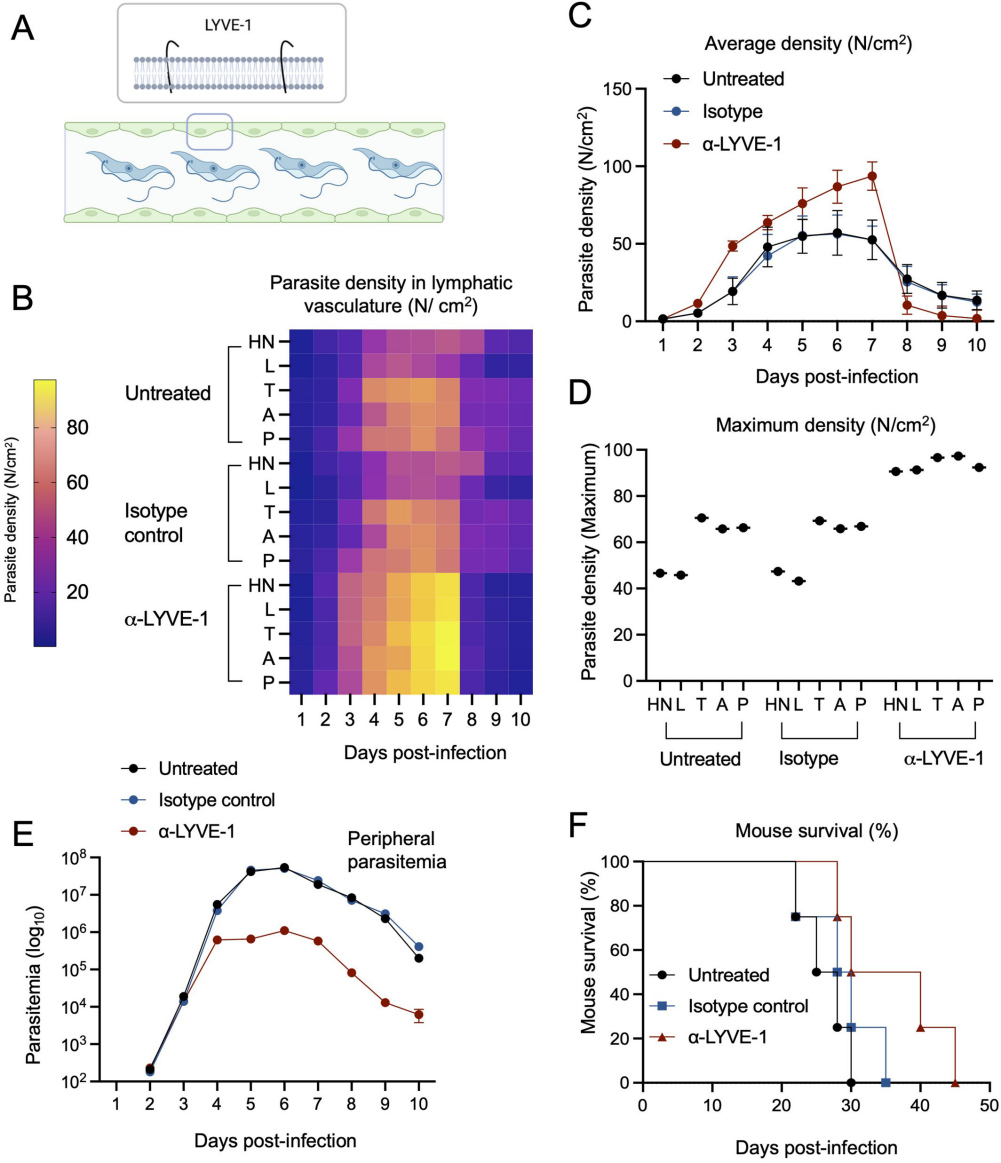
**The lymphatic vascular endothelial receptor (LYVE-1) plays an important role in parasite colonization of the lymphatic system and blood**. (A) Schematic representation of LYVE-1, a type I integral membrane glycoprotein present in the lymphatic vascular endothelium. (B,C) Parasite density (expressed as parasite number per area) was measured in the lymphatic vasculature of untreated mice, and mice treated with isotype control, and anti-LYVE-1 antibody to block the receptor. Parasite density was measured in lymphatic vasculature of the HN, L, T, A, P, anatomical regions. Heat map shows that anti-LYVE-1 treated mice reach higher parasite densities between days 4 and 7 post-infection in all anatomical locations, and lowest parasite densities between days 8 and 10. (C) Numerical representation of the heatmap shown in B. Untreated values are shown in black, isotype control in blue, and anti-LYVE-1 in red. Parasite density in the lymphatic vasculature of anti-LYVE-1-treated mice experiences drastic changes, with the highest maxima and the lowest minima compared to both control groups. (D) Parasite density maxima were significantly different between anti-LYVE-1-treated mice (93.6 parasites/cm^2^) and mice in both control groups (59 and 58.5 parasites/cm^2^). (E) Blood parasitemia was significantly impacted by treatment with anti-LYVE-1 blocking antibodies. Control groups (untreated and isotype control-treated) reached an average parasitemia of 1.46 and 1.5×10^7^ parasites/ml throughout 10 days of infection, with maxima of 5.4×10^7^ and 5.1×10^7^, respectively. Conversely, anti-LYVE-1-treated mice reached an average parasitemia of 3.4×10^5^ parasites/ml, with a maximum of 1.1×10^6^ parasites/ml. Dots show the average parasitemia at each day of infection. Error bars represent s.d. (F) Mouse survival was significantly impacted by anti-LYVE-1-treatment, with control groups surviving between 20 and 35 days post infection, and anti-LYVE-1-treated groups surviving between 30 and 45 days post-infection.

Based on our findings regarding host pathology and parasite survival related to the colonization of the lymphatic system, we went on to explore the effect of blocking the lymphatic vascular endothelial hyaluronan receptor 1 (LYVE-1), on parasite spread and survival, and on host peripheral parasitemia and survival (Fig. 7; Table S15). Methodological details are shown in Fig. S17. LYVE-1 is a type I integral membrane glycoprotein that contains hyaladherin and can bind hyaluronic acid, and is present as a cell surface receptor on lymphatic endothelial cells (Adamczyk et al., 2016; Jackson, 2019) (Fig. 7A). Given the large presence of *T. brucei* parasites in the lymphatic system, we used blocking and neutralizing antibodies to investigate the effect of the LYVE-1 receptor (or lack thereof) on parasite distribution and survival (Fig. 7A-D). For this purpose, we injected α-LYVE-1 antibody every 2 days to ensure the receptor was neutralised until day 10 post-infection. We observed that upon loss of available LYVE-1 receptor, parasite density displays a different dynamic to isotype-treated or untreated mice, with α-LYVE-1-treated mice displaying a sharp increase in parasitemia which was 2-fold higher than isotype-treated or untreated mice until day 7 post-infection. This was followed by a sharp loss in viability from days 8-10 post-infection (Fig. 7B,C). While the geometric mean of the global parasite density was not significantly different (Table S15), the maxima (reached between days 4 and 7 post-infection) (Fig. 7D), and minima (reached between days 8 and 10) were significantly different in all anatomical locations evaluated (i.e. HN, L, T, A, P) (*P*<0.05) (Table S15). The maximum parasite density in the lymphatic vasculature reached in untreated, isotype-treated and α-LYVE-1-treated mice was of 59, 58.4, and 82.3 parasites/cm^2^, respectively. The minimum parasite density (post-peak of parasitemia) in the lymphatic vasculature reached in untreated, isotype-treated and α-LYVE-1-treated mice was of 13.4, 12.9, and 4.3 parasites/cm^2^, respectively.

Based on this result in the lymphatic vasculature, we went on to investigate whether peripheral parasitemia (in the blood vasculature) was affected by α-LYVE-1-treatment (Fig. 7E; Table S16). We found that α-LYVE-1-treated mice have an overall 42.7-fold lower parasitemia than untreated mice, and 44-fold lower than isotype-control-treated mice (Table S16). Untreated and isotype-control-treated mice reached the peak of parasitemia at day 6 post-infection (5.4×10^7^ and 5.1×10^7^ parasites/ml, respectively). On the same day post-infection, parasitemia in α-LYVE-1-treated mice was 1.1×10^6^ parasites/ml. Although we stopped administration of the blocking antibody at day 10 post-infection, we went on to evaluate the effect of this treatment on mouse survival. We observed that untreated mice and isotype-control-treated mice lived between 20 and 35 days post-infection, while α-LYVE-1-treated mice lived between 30 and 50 days (Fig. 7F). This difference was significant (*P*=0.03) and suggests an important interplay between the lymphatic system and general homeostasis during *T. brucei* infection.

## DISCUSSION

The interplay between *T. brucei* and host lymph nodes has been an important focus of interest in *Trypanosoma* research since very soon after the discovery of the parasite by Sir David Bruce in 1894. Historical clinical and veterinary studies consistently describe lymph node palpation to detect enlargement as a key step in *T. brucei* diagnosis and follow-up (Boatin et al., 1986; Lutumba et al., 2005; Noireau et al., 1987; Nyimba et al., 2015; Vanhecke et al., 2010). Equally, lymph node aspirates allowing the isolation of *T. brucei* have been routinely performed for decades (Legros et al., 1999; Mumba Ngoyi et al., 2014; Scott et al., 1991). Around 100 studies published since the early 1900 s have addressed different aspects of the interaction between *T. brucei* and the host lymphatic system in multiple mammals including humans, non-human primates, horses, dogs, donkeys, sheep, rabbits, cattle, and experimental animal models including rats and mice. In these studies, the lymph node-*T. brucei* interplay has seen many directions. One of the main directions, is the haematological and immunological consequences of host infection, particularly, immune population changes in lymphoid organs (and ensuing changes in the periphery) of infected hosts (Baker and Taylor, 1971; Barry and Emergy, 1984; Brown and Losos, 1977; Gasbarre et al., 1981; 1980; Hu, 1934; Ikede et al., 1977; Ikede and Losos, 1972a,b; Luckins, 1972; Magez et al., 2002; 1997; McCully and Neitz, 1971; Moulton and Sollod, 1976; Murray et al., 1974; Murray et al., 1974b,a; Namangala et al., 2009; 2000b,a; Okomo-Assoumou et al., 1995; Olsson et al., 1991; Sileghem et al., 1989; van den Ingh, 1977). This has led to a wealth of information on naive and adaptive immune responses to, and the function of many immune cell types during *T. brucei* infection. Other directions that have been explored under the lymph node-*T. brucei* umbrella include lymph node pathology (Barrowman and Roos, 1979; Morrison et al., 1981), lymph node invasion and its importance for tissue distribution (Alfituri et al., 2020a,b; Rudin et al., 1984), and diverse variant antigen types in different anatomical locations of the host including the lymphatic system (Barry and Emergy, 1984; Seed and Effron, 1973).

Despite this wealth of work, several questions remain unanswered with regards to the relevance of *T. brucei* presence in the lymphatic system in terms of overall relevance to host pathology (beyond alterations to immune-system homeostasis); immune function and parasite recognition/elimination; and parasite heterogeneity. For the latter, it remains important to know whether the parasite population in the lymphatic system differs in any way from the parasite population in blood, and if it does, what the relevance of this difference is to infection and/or transmission. Between four and five decades ago, Ssenyonga and Adam, Hecker et al., and Tanner et al. (Hecker and Brun, 1982; Ssenyonga and Adam, 1975; Tanner et al., 1980) did the first (and to our knowledge, the only) morphological analysis of *T. brucei* in the lymph nodes. They showed for the first time that parasites in the lymph nodes had a morphology that was intermediate between the conventional slender and stumpy forms found in blood. They also proposed lymph nodes as important sites for parasite replication and antigenic variation. Taking this work as a basis, in our work, our main aim was to investigate whether parasite heterogeneity exists between 20 lymph node sets in the mouse and whether (and how) it differs from the *T. brucei* population in blood. We considered this study as relevant given findings done over the last decade on parasite tropism and motility (reviewed in Bargul et al., 2016; Crilly and Mugnier, 2021; Heddergott et al., 2012; Krüger et al., 2018; Krüger and Engstler, 2018; Silva Pereira et al., 2019), and the existence of modern tools allowed us to follow up the parasite populations with high spatial and temporal resolution (reviewed in De Niz et al., 2019a), previously not available.

We used *in vivo* and *ex vivo* microscopy in murine models of infection to investigate three key questions namely (a) are *T. brucei* parasites equally distributed across all lymph nodes, or are some lymph nodes more enriched than others, and how does this compare to blood? (b) Are *T. brucei* parasites different to those in blood in terms of stumpy/slender ratios, cell cycle progression, morphology and motility? And, (c) what is the impact of lymph node colonization on disease progression and, ultimately, mouse survival? We investigated all three questions at high-temporal resolution, given the characteristic cyclical pattern of infection, and reached conclusions that incorporate the time element as a key factor. We explore our findings in the context of each question below.

We found that parasite density in lymph nodes is different to that in blood both, in absolute numbers and considering temporal variations. The lymph node *T. brucei* population displays cyclical patterns that do not match those occurring in blood, showing a possible phase shift. We hypothesise that there is an interplay between the lymph node population and the blood population, with one affecting the other, although this remains a subject that demands further work in the future. While Ssenyonga and Adam suggested in 1975 (Ssenyonga and Adam, 1975) that a monomorphic trypanosome population is constantly flushed from the lymph to the blood, in our work we are unable to prove this hypothesis. However, the fact that a large live parasite population is present in the lymph nodes during parasitemia remission in the blood, and vice versa, suggests inter-organ communication that ensures parasite survival in separate anatomical locations. Our findings using LYVE-1-blocking antibodies, further support the possibility that the parasite population from either anatomical location is capable of replenishing the other. We found that blocking LYVE-1 resulted in an increased parasite burden in the lymphatic vasculature and decreased parasitemia. Mice treated with anti-LYVE-1 antibodies over a period of 10 days also survived longer than control mice. We hypothesise that LYVE-1 is an important receptor mediating parasite migration between the lymphatic system and other anatomical locations. An interesting follow-up question is to what extent reducing parasite migration from the lymphatic system impacts the establishment of well-known extravascular reservoirs such as those in the gonadal adipose tissue and the pancreas. Moreover, we hypothesise that this migration from the lymphatic vasculature is important for the overall survival of the parasite population, as the extended permanence of parasites in these compartments results in a sharp decline in parasite survival.

Also within the umbrella of this question, a further finding from our work is that parasite density and survival within different lymph node sets, is different. Namely, the lymph nodes located in the pelvis and abdomen are more heavily colonised than those in the limbs, head and neck, and thorax. Notably, our lab has previously mapped the largest extravascular reservoirs to organs in the pelvis and abdomen, i.e. the pancreas and gonadal adipose tissues (De Niz et al., 2021; Trindade et al., 2016). The abdominal and pelvic lymph nodes, together, help eliminate infections or inflammation in these regions, as well as drain inflammatory debris away from these regions to the systemic circulation. It is important to consider, however, that our work was based on intraperitoneal infections. While our previous work (De Niz et al., 2021) explored whether route of infection resulted in preferential colonization of some organs, and concluded that it did not, further investigation on this effect with respect to lymph nodes will be important to eliminate this as a potential confounder. Further work could look into molecular, cellular and biophysical factors (e.g. signalling, quorum sensing, and flows occurring between both organs, among others) mediating parasite migration between organs and lymph nodes.

Addressing our second question we found that the parasite population in the lymph nodes is significantly different from the one in blood. This is the case even considering major fluctuations through time of infection, observed across all factors analysed. Our findings are in agreement with previous reports (Hecker and Brun, 1982; Ssenyonga and Adam, 1975; Tanner et al., 1980), that defined the *T. brucei* lymph node population in the rat lymph nodes as a fast-replicating population with intermediate morphology. We here confirm that this is conserved in mice and is consistent across lymph nodes. Nevertheless, considering all factors analysed (replication rate, stumpy/slender ratios, area, length, width, and speed) differences across lymph nodes in the five anatomical locations analysed exist, with most factors being similar across the abdominal and pelvic lymph nodes on one hand, and across the head and neck, limbs and thorax lymph nodes on the other hand. Strikingly, though, our findings have important implications for our understanding of *T. brucei* survival, morphology and behaviour, showing that these factors are heavily influenced by the microenvironment in which the parasites are present. This is the focus of further work in our lab addressing other extravascular reservoirs. While Tanner et al. already raised the question of whether only a specific parasite population is capable of invading organs and tissues beyond the blood in 1980, this question remains unanswered in our work, and will be an important venue of our research in the future. Even more so in the current context of research aiming to understand how the overall *T. brucei* population is built, and the implications thereof (Beaver et al., 2023 preprint; Briggs et al., 2021). We found in our work, that different ‘intermediate’ parasite forms exist, not only in terms of morphology and speed, but also considering PAD1:GFP expression. This finding is extremely relevant, especially in the context of recent work that has led us to re-evaluate the paradigms of what stumpy and slender forms are, and the relevance of these to host-pathogen interactions including transmission (Briggs et al., 2021; Larcombe et al., 2023 preprint; Schuster et al., 2021).

It remains to be understood why parasites in lymph nodes are generally bigger and faster than parasites in blood (despite major fluctuations with time possibly linked to parasite replication and influx/efflux across organs and blood). Previous work has suggested that faster swimmers are better able to eliminate host antibodies (Engstler et al., 2007). Therefore, a possible hypothesis is that faster movement in lymph nodes is necessary for parasite survival. However, other alternatives exist including escape from immune cells, response to alternative signals present in the lymph nodes but not the blood, or the presence of exogenous components stimulating movement including the effect of forces acting on lymph flow. It is possible to hypothesise that both, host and parasite factors are involved in regulating parasite adaptation. Biophysical and biomechanical components in the two major tissues investigated in this work are largely different in terms of extracellular matrix, collagen presence, viscoelasticity, and the presence and nature of flow related to blood and lymphatic transport (Magno et al., 2015; O'Melia et al., 2019; Rohner et al., 2015; Wiig and Swartz, 2012). Other possible factors include those related to infection itself, such as changes in viscosity and temperature. Whether and how these parameters might influence parasite adaptation, remains an open question. Additional to this important point, another observation arising from this work worth following up was the relatively low proportion of stumpy forms found in the lymph nodes. Whether this arises because stumpy forms are incapable of invading lymph nodes, or because lymph nodes do not support differentiation, or stumpy form survival, this finding has important implications for our understanding of *T. brucei* transmission. So far, the field is most familiar with slender/stumpy fluctuations in the blood, but we know very little about these fluctuations at other organ and sub-organ levels. Our findings here add to the large wealth of work being generated to understand the relevance of tissue reservoirs for overall parasite survival and transmission potential.

Finally, in response to our third question, we found that both, lymph nodes and the lymphatic vasculature are highly remodelled throughout infection. While we have shown that remodelling of equivalent nature occurs in other organs (De Niz et al., 2021; Machado et al., 2023; Trindade et al., 2016), it is important to consider the implications of lymphatic system compromise. Although *T. brucei* is not considered *per se* a lymphatic disease as lymphatic filariasis is, it induces remarkable changes in systemic homeostasis as shown by the presence of major oedema (composed of heavily parasitised fluid) throughout the whole mouse body. This suggests alterations in lymphatic transport at a whole body level, which might have important implications for individual organ homeostasis (reviewed in Stritt et al., 2021) and overall immune function (reviewed in Liao and Padera, 2013), to name some factors. Altered lymphatic flow has been the subject of study in multiple other diseases (Cueni and Detmar, 2008; Liao and Padera, 2013; Mallick and Bodenham, 2003; Padera et al., 2016; Thomas et al., 2016) and is certainly an exciting new venue of research for *T. brucei*.

Altogether, we have found that lymph nodes harbour a unique population of *T. brucei* parasites which display significant fluctuations related to infection progression – suggesting adaptation processes similar to those occurring in the blood. Moreover, we show that while lymphatic system alterations have not been the focus of studies aiming to understand overall host pathology related to nagana or Human African trypanosomiasis, they might be key to our understanding of the big picture of host–pathogen interactions, immune response to *T. brucei,* and overall host pathology resulting from possible lymphadenopathy. Our work on lymph nodes is complementary to our studies of parasite heterogeneity in other extravascular sites, currently in progress. We are sure these findings pose an exciting basis for future research on *T. brucei* heterogeneity, parasite transmission, immunopathology and host-parasite biophysics and biomechanics.

## MATERIALS AND METHODS

### Animals models

Animal experiments were performed according to EU regulations and approved by the Animal Ethics Committee of Instituto de Medicina Molecular (IMM) (AWB_2016_07_LF_Tropism 018889/2016). Mice used across this study included wild-type C57BL/6J mice obtained from Charles River, France. Breedings were generated in-house at IMM. All mice were 6-9-week-old males and females, with an average weight ranging between 25 and 30 g.

### Trypanosoma brucei parasites and infections

Mice were infected by intraperitoneal injection of either 3000* T. brucei* AnTat 1.1^E^ chimeric triple reporter parasites (Calvo-Alvarez et al., 2018) expressing the red-shifted firefly luciferase protein PpyREH9, TdTomato and Ty1 or GFP::PAD1-expressing *T. brucei* AnTat 1.1^E^. Based on our previous findings on the time of highest disease recrudescence in the survival experiments (De Niz et al., 2021), most experiments were capped to 20 days of infection, except those relevant to terminal body fluid and oedema measurements. Experiments relative to terminal body fluid were done with infections of 2000* T. brucei* EATRO1125 AnTat 1.1E 90-13 parasites.

### Intravital and *ex vivo* imaging

For intravital imaging, surgeries were performed as described in De Niz et al., 2019b, 2019c, 2019d, 2020 for the various anatomical locations. Briefly, mice were anaesthetised with a mixture of ketamine (120 mg/kg) and xylazine (16 mg/kg) injected intraperitonially. Following verification of lack of reflex, mice were intraocularly injected with Hoechst 33342 (stock diluted in dH_2_O at 100 mg/ml; injection of 40 μg/kg mouse), 70 kDa FITC-Dextran (stock diluted in 1x PBS at stock concentration of 100 mg/ml; injection of 500 mg/kg), and LYVE-1 conjugated to A647 (clone 223322 R&D systems, used at 20 μg). A temporary glass window (Merk rectangular coverglass, 100 mm×60 mm or circular coverglass, 12 mm) was implanted in each organ, and secured either surgically, with surgical glue, or via a vacuum, in order to enable visualization of the lymph node surface. All imaging was done in a Zeiss Cell Observer SD (spinning disc) confocal microscope (Carl Zeiss Microimaging, equipped with a Yokogawa CSU-X1 confocal scanner, an Evolve 512 EMCCD camera and a Hamamatsu ORCA-flash 4.0 VS camera) or in a 3i Marianas SDC (spinning disc confocal) microscopy (Intelligent Imaging Innovations, equipped with a Yokogawa CSU-X1 confocal scanner and a Photometrics Evolve 512 EMCCD camera). Laser units 405, 488, 561 and 640 were used to image Hoechst in nuclei, draining lymph nodes and lymphatics marked by FITC-Dextran and GFP::PAD1 in *T. brucei*, TdTomato in *T. brucei*, and LYVE1 in the lymphatic vascular endothelium, respectively. The objective used to image parasite density and vascular morphology was a 40x LD C-Apochromat corrected, water immersion objective with 1.1 NA and 0.62 WD. The objective used to determine all cell cycle, survival, morphological and behavioural characteristics of *T. brucei* was a 100x Plan-Apochromat, oil immersion objective with 1.4 NA and 0.17 WD. Between 20 and 100 images were obtained in any one time lapse, with an acquisition rate of 20 frames per second. Since not all lymph nodes were easily accessible by intravital surgery, in order to gain access to all lymph node sets, we performed *ex vivo* imaging by excising the lymph node. For this, we performed z stacks consisting of 16 stacks covering up to 200 μm of tissue depth. For all acquisitions, the software used was ZEN blue edition v.2.6 (for the Zeiss Cell Observed SD) allowing export of images in .czi format, and 3i Slidebook reader v.6.0.22 (for the 3i Marianas SD), allowing export of images in TIFF format.

### Lymph node identification and measurement

Lymph nodes were identified as described by Van den Broeck et al. (Van den Broeck et al., 2006) and Harrell et al. (Harrell et al., 2008), by injection of subcutaneous, intrahepatic, and intradermal injection of Evans Blue dye. Result were confirmed by injection of 70 kDa FITC-Dextran whenever intravital microscopy was possible. The initial characterization was performed in infected mice at day 10 post-infection, when lymph nodes were enlarged, ensuring their correct identification from surrounding tissue. Confirmation of correct lymph node identification was performed by microscopy and comparison of anatomical location to the detailed mapping described by Van den Broeck (Van den Broeck et al., 2006).

### Parasite density and diameter quantification

In order to quantify parasite density, we considered the total area of the field of view in the lymph nodes, and quantified the number of parasites in such area. Results shown in Tables S1 and 2 are the average of at least 100 measurements in at least 50 separate fields of view in three mice. To quantify parasite density in the lymphatic vasculature, for normalization of parasite load (i.e. expression as parasites per cm^2^ of vessel), we took as reference the vascular marker LYVE1-A647. We segmented the area demarcated by LYVE.1, and quantified parasites within this area. We then expressed parasite number as a function of the area. Results are shown in Table S13. Vascular density measurements were performed throughout 20 days of infection. Vessel diameters and areas were measured using Fiji (Schneider et al., 2012). To identify the lymphatic vasculature, we intravenously injected 20 μg of antibodies against LYVE-1 (BioLegend) conjugated to A647, into infected mice at each day of infection, as previously described in the context of parasitology for CD31 (De Niz et al., 2021; 2018; Hopp et al., 2015). We also intravenously injected 20 μg of antibodies against CD31 conjugated to A647, for blood vessel identification.

### Quantification of parasite survival, cell cycle progression and stumpy:slender ratios

Parasite survival was defined by two features: absolute absence of parasite motility (not only lack of displacement but also lack of flagellar beating) and markers of cell death including blebbing or parasite destruction. Results shown in Table S3 are the percentage of live parasites in at least 50 fields of view in three independent mice, measured throughout 20 days of infection. Cell cycle progression was characterised by intravenous injection of Hoechst 33342 – this results in the labelling of the parasite nucleus (N) and kinetoplast (K), allowing for their quantification. Parasites were classified into three groups: 1 kinetoplast, 1 nucleus (1K1N), 2 kinetoplasts, 1 nucleus (2K1N), and 2 kinetoplasts, 2 nuclei (2K2N). Results shown in Table S4 are the percentage of live parasites in at least 50 fields of view in three independent mice, measured throughout 20 days of infection. While all other experiments were performed using a TdTomato reporter line, stumpy/slender ratios were measured by the use of a GFP::PAD1 line, in which GFP is expressed in parasites differentiating into stumpy forms or those fully differentiated. While we made a sub-classification in our work, this is included as Fig. S6. For this reason, most results are expressed as proportion of true slender forms (i.e. those in which no GFP expression was detected and morphological characterization excluded a stumpy phenotype). Results shown in Table S5 are the percentage of true slender forms (non-GFP-expressing and with non-stumpy morphology) in at least 50 fields of view and three independent mice, measured throughout 20 days of infection.

### Morphological and behavioural analysis

Morphological analyses were performed using Fiji (Schneider et al., 2012). Parasite width was calculated in the TdTomato reporter *T. brucei* line by measuring parasite diameter at the widest point (close to the location of the nucleus/nuclei). Parasite length was measured from the flagellar tip to the parasite anterior (from edge to edge). While in some cases the full area of the parasite was visible this was not always the case. In order to avoid confounding resulting from the parasite's positioning, we calculated area for all parasites as the proxy of width by length. This will in all cases be slightly higher than the true area value, which varies based on the proportion of the free flagellum. Results shown in Tables S6, 7 and 8 are the average area of at least 50 parasites in at least three independent mice measured throughout 20 days of infection. Behavioural analysis was limited to the measurement of parasite speed. Between 20 and 100 images were obtained in any one time lapse, with an acquisition rate of 20 frames per second. Speed was calculated as the function of distance over time. Results shown in Table S9 are the average speed of at least 50 parasites in at least three independent mice measured throughout 20 days of infection.

### Quantification of oedema-related parasitemia

In infected mice, once oedemas began to form, free fluid could be easily acquired. At least 1 µl of fluid was obtained from the abdominal-pelvic or thoracic region of the mice and diluted in 200 µl of 1x PBS. Parasite load was quantified using disposable hemocytometers. Results shown in Table S14 are the average parasitemia in three independent mice measured throughout 20 days of infection for animals infected with the TdTomato reporter line. For animals where full longitudinal studies were conducted, fluid was collected, and parasite burden analysed at the point of death in six mice infected with WT *T. brucei.* This was on days 33, 36 (2x), 39, 40, and 51 post-infection.

### Measurements of percentage of body fluid mass

Free fluid mass was determined using a 6.2 MHz time-domain nuclear magnetic resonance small animal body composition analyser (Minispec LF65, Bruker). Data were normalised to free fluid of mice prior to infection.

### Blocking lymphatic endothelial receptor LYVE-1

To investigate the effects of blocking LYVE-1, we used the antibodies MAB2125 (R&D systems, 20 μg per mouse), and Mouse IgG2 isotype controls were used as controls (eBioscience). Antibodies were injected intravenously every 2 days by tail vein injection, starting on the day of infection, and continuing until day 10 post-infection.

### Hierarchical clustering analysis

Hierarchical clustering analysis was performed using the Heatmapper tool developed by Babicki et al. (Babicki et al., 2016) available at http://heatmapper.ca. Dendrograms and heatmaps were generated using the Expression tool. For all cases we used average linkage as the clustering method, and Euclidean distance measurement method. The distance and correlation matrices in Fig. 4 were generated using the Pairwise tool, also based on Euclidean distance measurement.

### Value normalization and feature selection

Data normalization (shown in Table S10) was performed for all data shown in Table S1 and 3-9 using the formula (x−x)/s where x is the data value (the global average of each criteria), 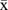 is the mean of the dataset (considering all organs analysed), and s is the standard deviation of the dataset. The normalised value shows how many standard deviations each datapoint is from the mean, and whether it is greater (if the value is above 0) or less (if the value is below 0) than the mean. Feature selection was done by calculating the mean absolute deviation (MAD), which can be obtained by the formula *MAD*=*median*(|*x*_*i*_−*x*_*m*_|). *x*_*i*_ is the i^th^ value in the dataset, while *x*_*m*_ is the median value in the dataset. While feature selection can be used to discard features in a complex analysis, in our work we used this analysis to highlight the most significant factors for the distinction between LN and blood *T. brucei* populations.

### Quantification and statistical analysis

Data were displayed in graphs and heatmaps generated using Prism 9 software (GraphPad). Means, medians, survival, correlation tests, comparison tests, and error measures were calculated from triplicate experiments with three biological replicates each, and/or at least 100 images per condition. Means or geometric means were calculated for each section, as appropriate. For comparisons of measurements between LN and LN groups we performed multiple *t*-tests in addition to a one-way ANOVA (differences were considered significant when *P*<0.05). Pearson correlations measures (R), and R^2^ values were calculated to determine the strength of linear association between various parameters. For comparisons of survival, a log-rank (Mantel-Cox) test was performed, and *P*-values<0.05 were considered significant. Statistical details of experiments are included in the figure legends, the results section, and a Table Sexcel file. All data used for the generation of the figures is included as a supporting file.

## Supplementary Material

10.1242/biolopen.059992_sup1 Supplementary information

Table S1. Parasite density (related to Figure 1B)

Table S2. Parasite density (related to Figure 1C)

Table S3. Parasite survival (related to Figure 1E and 1F)

Table S4. Percentage of 1K1N (related to Figure 2A and 2B)

Table S5. Percentage of slenders (related to Figure 2D and 2E)

Table S6. Parasite width (related to Figure S2A)

Table S7. Parasite length (related to Figure S2B)

Table S8. Parasite area (related to Figure 3A-3B)

Table S9. Parasite speed (related to Figure 3C-3D)

Table S10. Normalized values used for Figures 4A and 4B

Table S11. MAD scores for Figure 4C

Table S12. Lymph node growth for Figure 5A and 5B

Table S13. Lymphatic vessel dilation for Figure 5C,D,E

Table S14. Parasitemia in oedemas and free fluid: Figure 6

Table S15. LYVE-1 blocking effect on parasite density in lymphatic vessels - Figure 7A- 7C

Table 16. LYVE-1 blocking effect on parasite density in blood vessels - Figure 7D-7F
