# Supplementary material for: The lymphatic system favours survival of a unique T. brucei population
Source: Biol Open. 2023 Nov 9;12(11):bio059992. doi: 10.1242/bio.059992 (PMC10651106; doi:10.1242/bio.059992)
Supplement: Supplementary information [file biolopen-12-059992-s1.pdf]

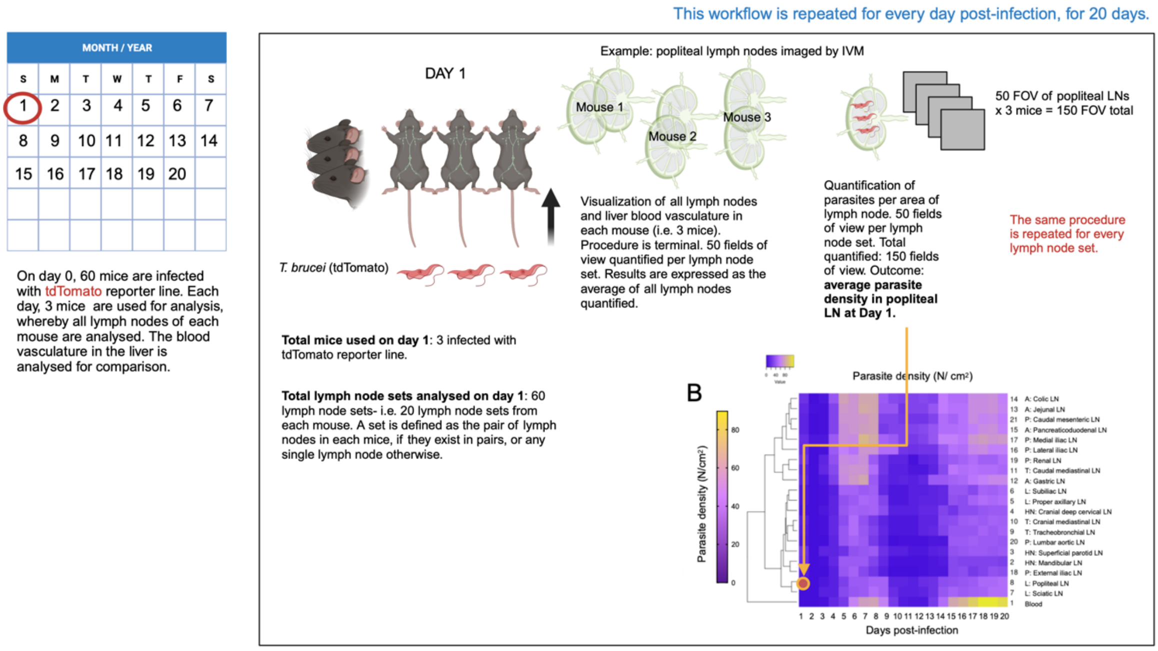


**Fig. S1. Methodological detail corresponding to Figure 1B-1C.** 60 mice were infected on day 0, with 3000 parasites of the AnTat 1.1E chimeric triple reporter line expressing red-shifted firefly luciferase, tdTomato and Ty1. Parasites were injected intraperitoneally. Every day, starting at day 1 post-infection, 3 mice were used and 20 lymph node sets of each mouse were analysed. The schematic shown in this figure shows the workflow corresponding to day 1 in the popliteal lymph node as an example. 50 fields of view of each lymph node set in each mouse were used for quantification, giving a total of 150 fields of view. The average parasite density in each lymph node set of each of the 3 mice (i.e. 150 fields of view) was used for each time point, for each lymph node set. The arrow points to the value that the workflow corresponding to the popliteal lymph node at day 1, leads to. 60 lymph node sets (20 lymph node sets per mouse) are explored each day. Quantifications are the result of average of 3 mice per day for each lymph node set.

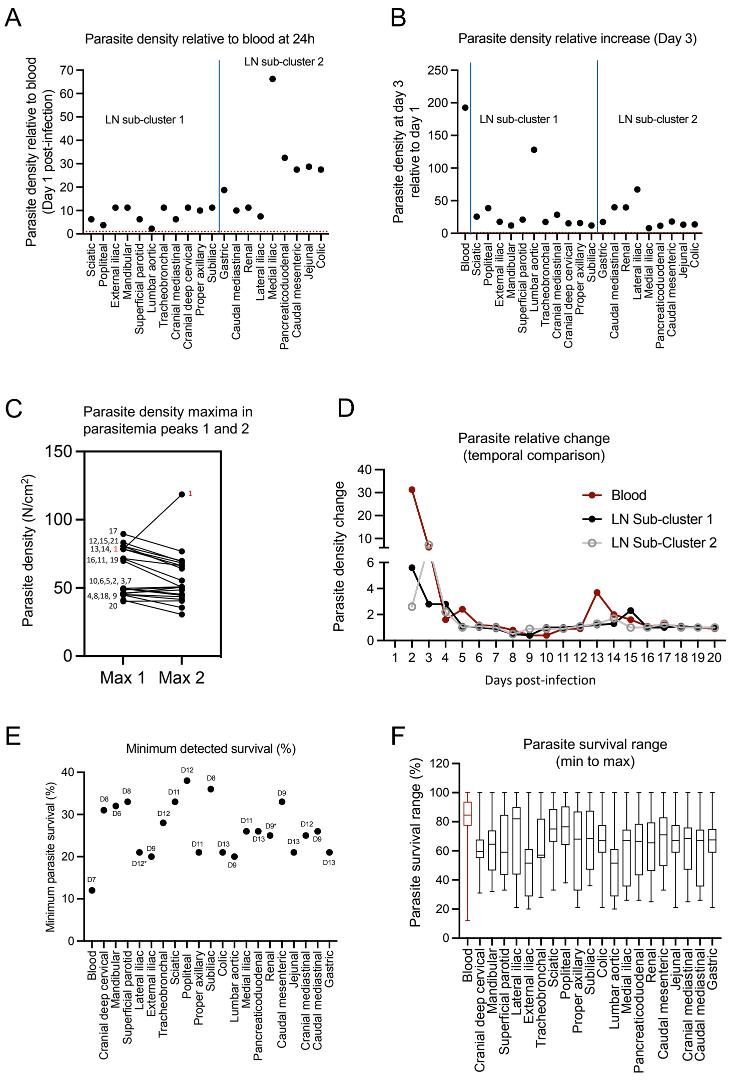


**Fig. S2. Analysis at high temporal resolution showed that during the first 24 hours after infection all lymph nodes are colonized faster than blood. A)** 24 hours after infection they have, on average 8.2-fold (sub-cluster 1) and 25.5-fold (sub-cluster 2) higher parasite densities than blood (shown as a red dotted line). **B)** Parasite density in the blood rises quickly, rising by 192.5-fold by day 3 post-infection compared to day 1, while lymph nodes in sub-cluster 1 and sub-cluster 2 reach a relative increase of 30.1-fold and 25.4-fold respectively. **C)** The maximum parasite density across all days is reached in the second peak of parasitemia in the blood, but not in most lymph nodes. In most lymph nodes, the absolute maximum occurs during the first peak of parasitemia. Yet the difference between the maximum density at the first and second peaks of parasitemia is non-significant. **D)** The early stage of infection and the remission phase seem to favour lymph node invasion over blood, and throughout all 3 infection stages parasite density variations in the lymph nodes remain minimal. **E)** Although on average survival was highest in the blood, an absolute minimum of 12% survival was recorded in the blood, while the minimum survival in lymph node sub-clusters 1 and 2 was of 29.2 and 24.4% respectively. **F)** Survival fluctuations were highest in the blood compared to lymph nodes.

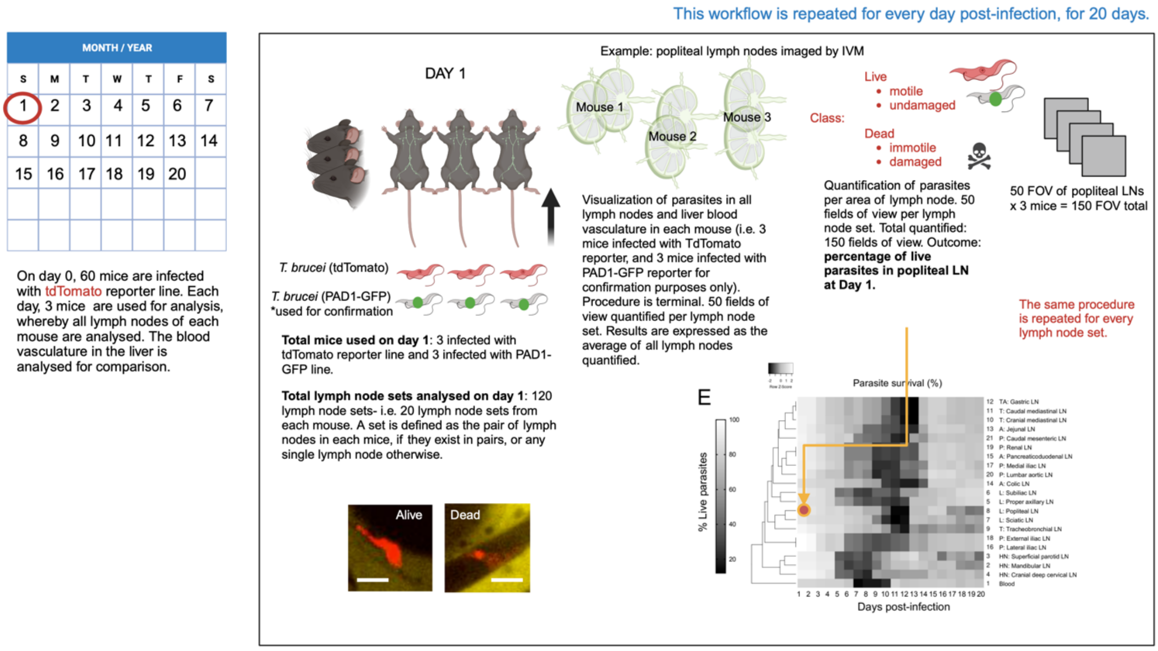


**Fig. S3. Methodological detail corresponding to Figure 1E-1F.** 60 mice were infected on day 0, with 3000 parasites of the AnTat 1.1E chimeric triple reporter line expressing red-shifted firefly luciferase, tdTomato and Ty1. Parasites were injected intraperitoneally. Every day, starting at day 1 post-infection, 3 mice were used and 20 lymph node sets of each mouse were analysed. The schematic shown in this figure shows the workflow corresponding to day 1 in the popliteal lymph node as an example. 50 fields of view of each lymph node set in each mouse were used for quantification, giving a total of 150 fields of view. In parallel, 60 mice were infected on day 0, with 3000 parasites of the PAD1:GFP reporter line. The percentage of live parasites in each lymph node set of each of the 3 mice (i.e. 150 fields of view) was used for each time point, for each lymph node set. The arrow points to the value that the workflow corresponding to the popliteal lymph node at day 1, leads to. 60 lymph node sets (20 lymph node sets per mouse) are explored each day. Quantifications are the result of averages of 3 mice per day for each lymph node set. Inset images show a live parasite, and a dead parasite displaying blebbing and body fragmentation. Scale bar 10 µm.

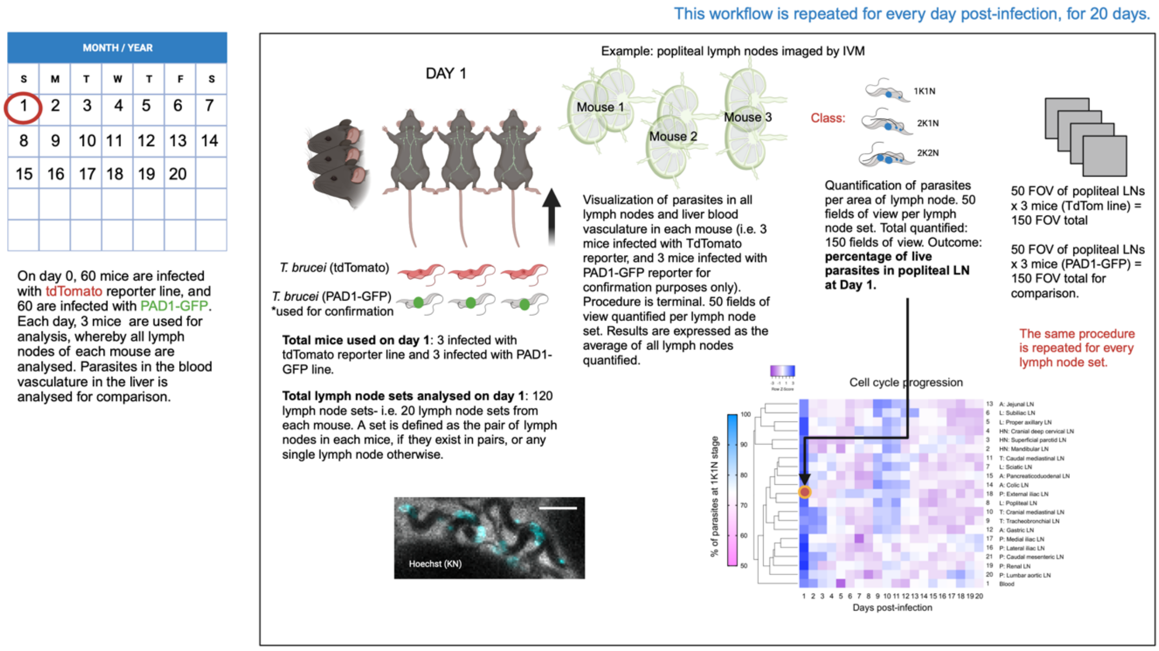


**Fig. S4. Methodological detail corresponding to Figure 2A-2B.** 60 mice were infected on day 0, with 3000 parasites of the AnTat 1.1E chimeric triple reporter line expressing red-shifted firefly luciferase, tdTomato and Ty1. Parasites were injected intraperitoneally. Every day, starting at day 1 post-infection, 3 mice were used and 20 lymph node sets of each mouse were analysed. The schematic shown in this figure shows the workflow corresponding to day 1 in the popliteal lymph node as an example. 50 fields of view of each lymph node set in each mouse were used for quantification, giving a total of 150 fields of view. In parallel, 60 mice were infected on day 0, with 3000 parasites of the PAD1:GFP reporter line. The percentage of parasites at 1K1N, 2K1N, 2K2N was used for each time point, for each lymph node set. The arrow points to the value that the workflow corresponding to the popliteal lymph node at day 1, leads to. 60 lymph node sets (20 lymph node sets per mouse) are explored each day. Quantifications are the result of averages of 3 mice per day for each lymph node set. Inset images show example of Hoechst staining of kinetoplasts and nuclei in multiple extravascular parasites. Scale bar 10 µm.

Biology Open • Supplementary information

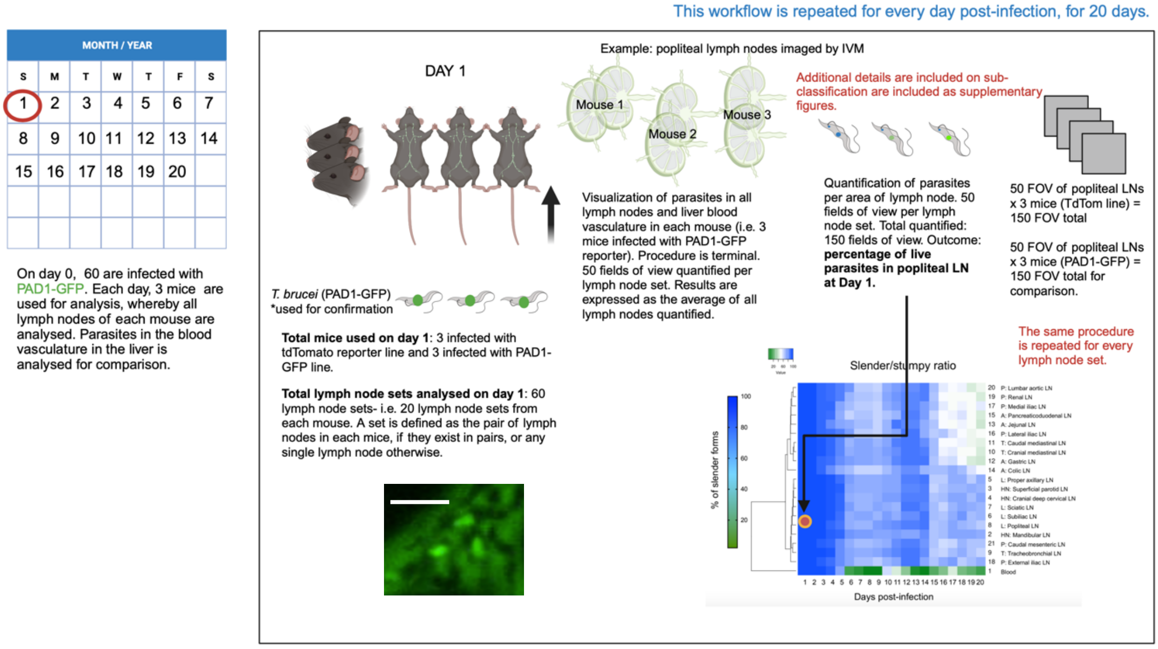


**Fig. S5. Methodological detail corresponding to Figure 2D-2E.** 60 mice were infected on day 0, with 3000 parasites of the PAD1:GFP reporter line. Parasites were injected intraperitoneally. Every day, starting at day 1 post-infection, 3 mice were used and 20 lymph node sets of each mouse were analysed. The schematic shown in this figure shows the workflow corresponding to day 1 in the popliteal lymph node as an example. 50 fields of view of each lymph node set in each mouse were used for quantification, giving a total of 150 fields of view. The percentage of parasites expressing GFP was used for each time point, for each lymph node set. A sub-classification of parasites based on other morphological features is shown in figure S6. The arrow points to the value that the workflow corresponding to the popliteal lymph node at day 1, leads to. 60 lymph node sets (20 lymph node sets per mouse) are explored each day. Quantifications are the result of averages of 3 mice per day for each lymph node set. Inset images show example of PAD1:GFP tagging in multiple extravascular parasites. Scale bar 20 µm.

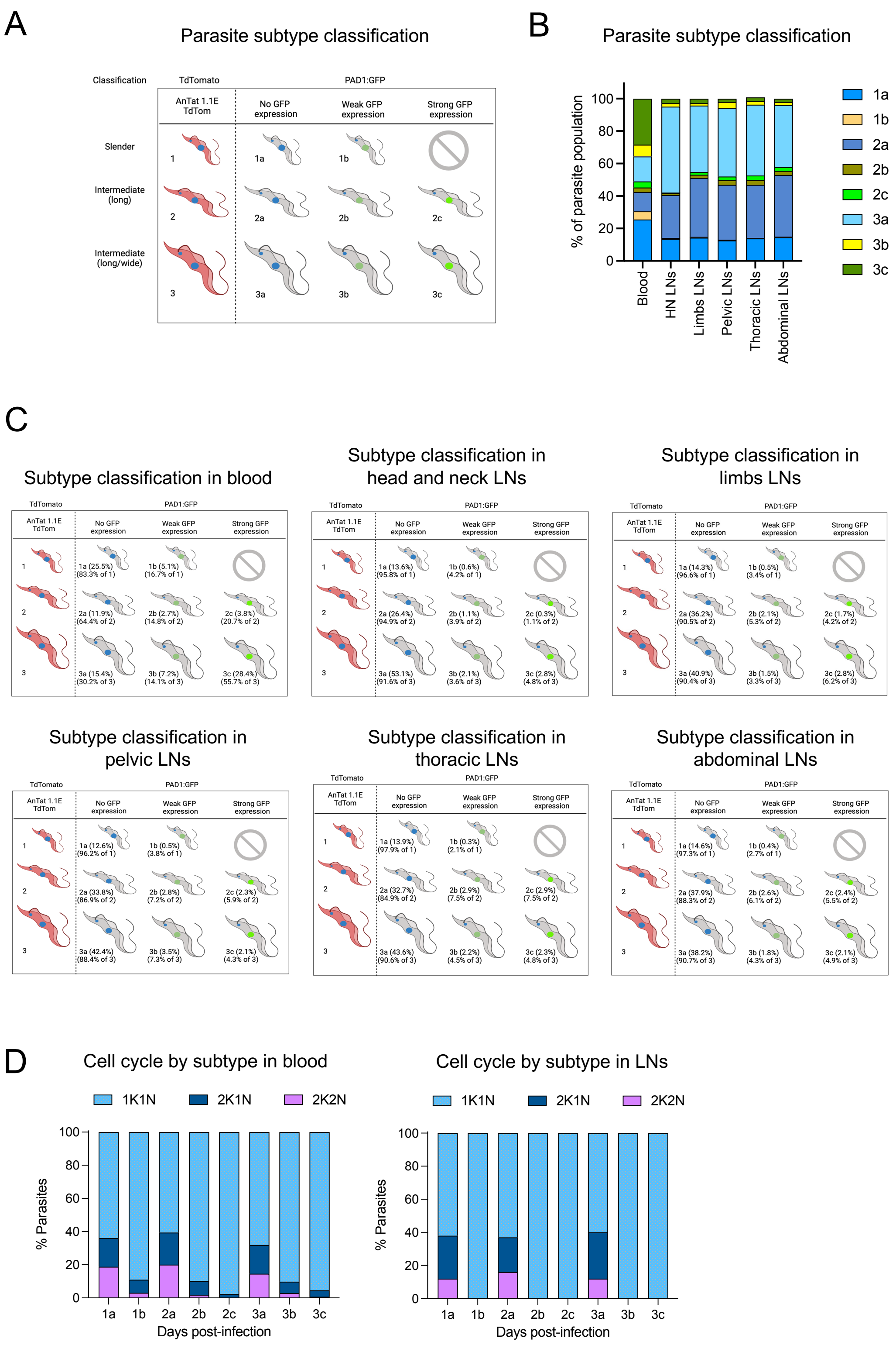


**Fig. S6. Various intermediate parasite forms exist in the blood and lymph nodes.** A) As we observed many non-stumpy forms expressing PAD1:GFP, we sub-classified these parasites based on their length and width. We found that slender forms with morphological characteristics similar to those observed in *in vitro* cultures existed without expression of GFP (class 1a), and with

intermediate expression of GFP (with less than 50% fluorescence intensity values compared to fully differentiated stumpy forms in culture). These were classified as "weak GFP expression slender forms" (class 1b). In the tdTomato reporter line, both sub-classes belong to class 1. Parasites showing over 12% increase in length compared to class 1 parasites, but less than 10% increase in width were classified as class 2 in the tdTomato reporter line corresponding to "intermediate long" forms. Within this class, 3 sub-classes were found using the PAD1:GFP reporter line. Namely, parasites with no PAD1:GFP expression (class 2a); parasites with weak PAD1:GFP expression (class 2b); and parasites with PAD1:GFP expression whereby GFP fluorescence intensity was similar to the one detected in fully differentiated stumpy forms (class 2c). Parasites showing over 12% increase in length compared to class 1 parasites, and over 10% increase in width were classified as class 3 in the tdTomato reporter line corresponding to "intermediate long/wide" forms. Within this class, 3 sub-classes were found using the PAD1:GFP reporter line. Namely, parasites with no PAD1:GFP expression (class 3a); parasites with weak PAD1:GFP expression (class 3b); and parasites with PAD1:GFP expression whereby GFP fluorescence intensity was similar to the one detected in fully differentiated stumpy forms (class 3c). **B-C)** Parasite sub-classification per anatomical location pooling all lymph nodes of such location. Figure S6B is the summary of figure S6C. Figure S6C shows the percentage of each parasite sub-class in the blood (top left panel), lymph nodes of the head and neck (top middle panel), lymph nodes of the limbs (top right panel), lymph nodes of the pelvis (bottom left panel), lymph nodes of the thorax (bottom middle panel), and lymph nodes of the abdomen (bottom right panel). Each panel shows the percentage of each sub-class among all parasites (top line), and the percentage of each sub-class per class (e.g. percentage of 1a within class 1). Figure S6B shows that the blood is enriched with sub-classes 1a (25.5% of all parasites) and 3c (28.4% of all parasites). Conversely, lymph nodes are enriched in parasite sub-classes 2a (ranging between 26.4 and 37.9% of all parasites), and 3a (ranging between 38.2 and 53.1% of all parasites). **D)** Cell cycle analysis of each parasite sub-class shows that in both, blood and lymph nodes, parasites of sub-classes 1a, 2a, and 3a (i.e. all those of the PAD1:GFP parasite line not expressing GFP) show enrichment of 2K1N and 2K2N stages, while sub-classes 1b, 2b, 2c, 3b and 3c show little to no 2K1N and 2K2N stages, suggesting that these sub-classes are cell-cycle arrested, albeit sharing morphological characteristics with the "a" parasite sub-class.

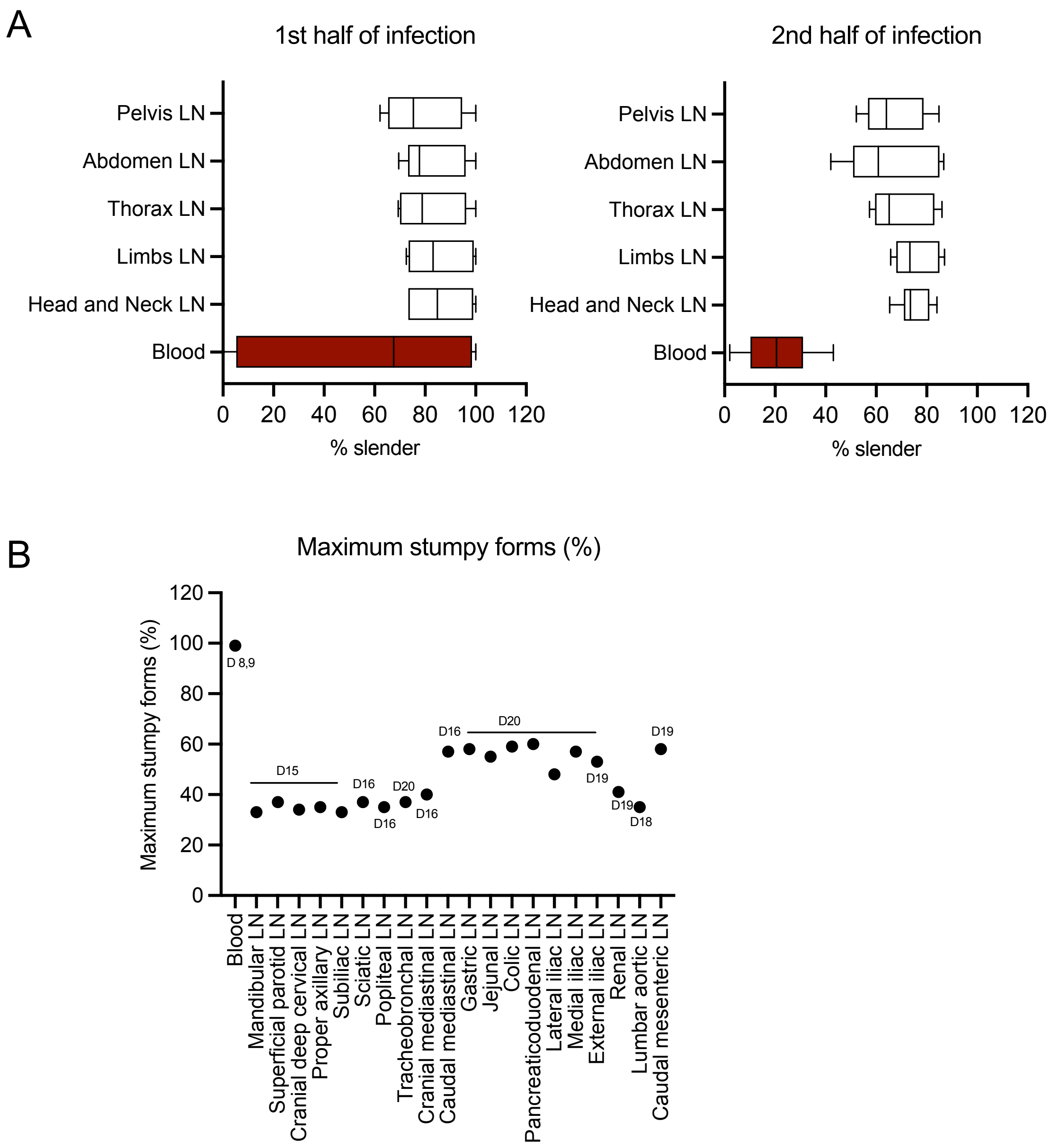


**Fig. S7. The lymph nodes are prohibitive for stumpy formation.** A-B) During the first half of the infection, the average slender percentage in blood is 54.6%, while in the second half it is 21.3% reaching a minimum of 2%. In lymph node cluster 1 (HN and L), slender presence remains significantly higher throughout infection, with an average of 85.7% during the first 10 days, 74.9% during the last 10 days, and a minimum of 63% at any point during the infection. In lymph node cluster 2 (T, A and P), slender presence remains significantly higher than in blood throughout infection, with an average of 81.3% during the first 10 days, 67.4% during the last 10 days, and a minimum of 40% at any point during the infection (between days 16 and 20).

Biology Open • Supplementary information

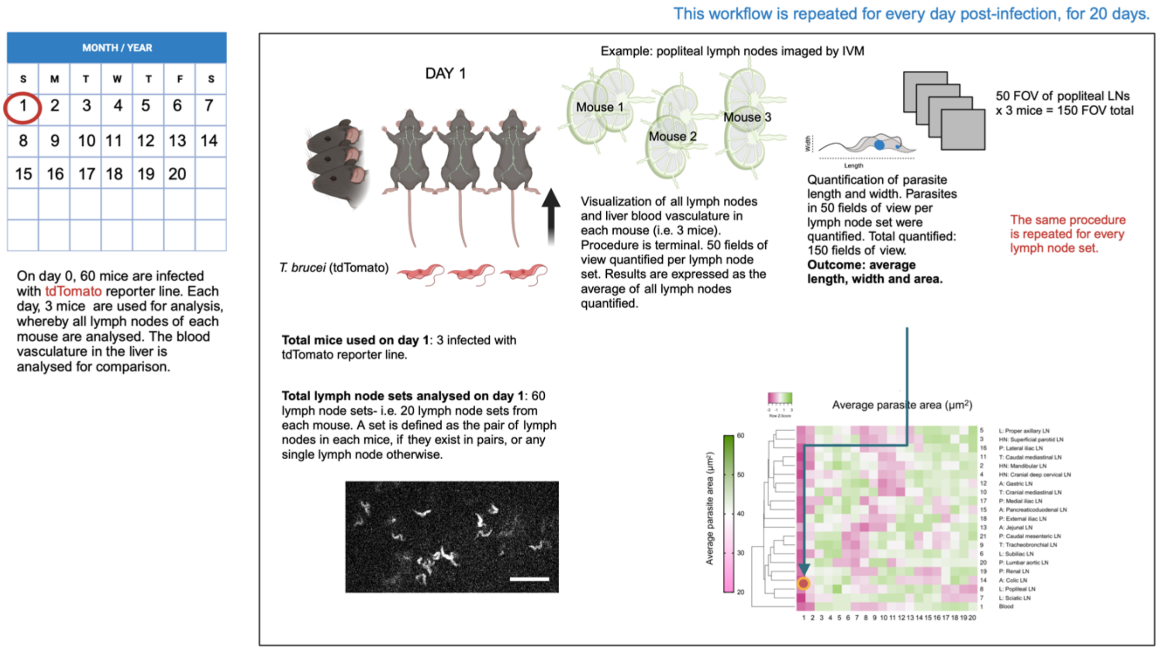


**Fig. S8. Methodological detail corresponding to Figure 3A and 3B.** 60 mice were infected on day 0, with 3000 parasites of the AnTat 1.1E chimeric triple reporter line expressing red-shifted firefly luciferase, tdTomato and Ty1. Parasites were injected intraperitoneally. Every day, starting at day 1 post-infection, 3 mice were used and 20 lymph node sets of each mouse were analysed. The schematic shown in this figure shows the workflow corresponding to day 1 in the popliteal lymph node as an example. 50 fields of view of each lymph node set in each mouse were used for quantification, giving a total of 150 fields of view. A minimum of 100 parasites were quantified in total. The parasite area was calculated from the length and width of each parasite. The arrow points to the value that the workflow corresponding to the popliteal lymph node at day 1, leads to. 60 lymph node sets (20 lymph node sets per mouse) are explored each day. Quantifications are the result of averages of 3 mice per day for each lymph node set. Inset image shows multiple parasites with a wide range of morphologies. Scale bar: 30 μm.

Biology Open • Supplementary information

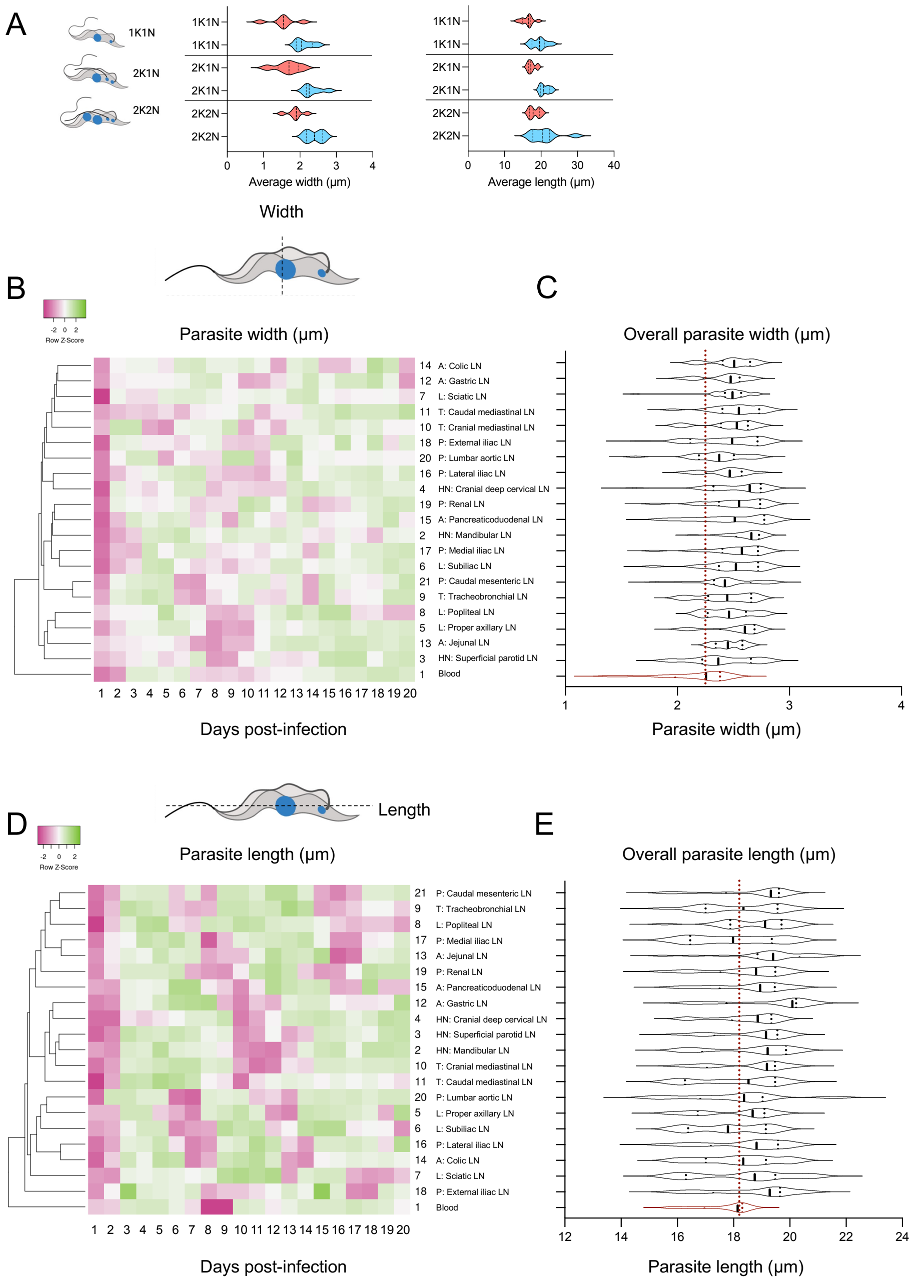


**Fig. S9. The *T. brucei* population in the lymph nodes is signifcantly longer and wider than the parasite population in the blood.** A) Average parasite width and length by cell cycle stage in blood and lymph nodes. Results are the pool of at least 100 parasites across all days of infection. B) Hierarchical clustering for width measurements showed the blood as a separate cluster to all lymph nodes, with no other significant clusters between the lymph node sets. C) Violin plots showing width distribution measurements for parasites in blood (red) and lymph nodes. D) Hierarchical clustering for length measurements showed the blood as a separate cluster to all lymph nodes, with no other significant clusters between the lymph node sets. E) Violin plots showing length distribution measurements for parasites in blood (red) and lymph nodes.

Biology Open • Supplementary information

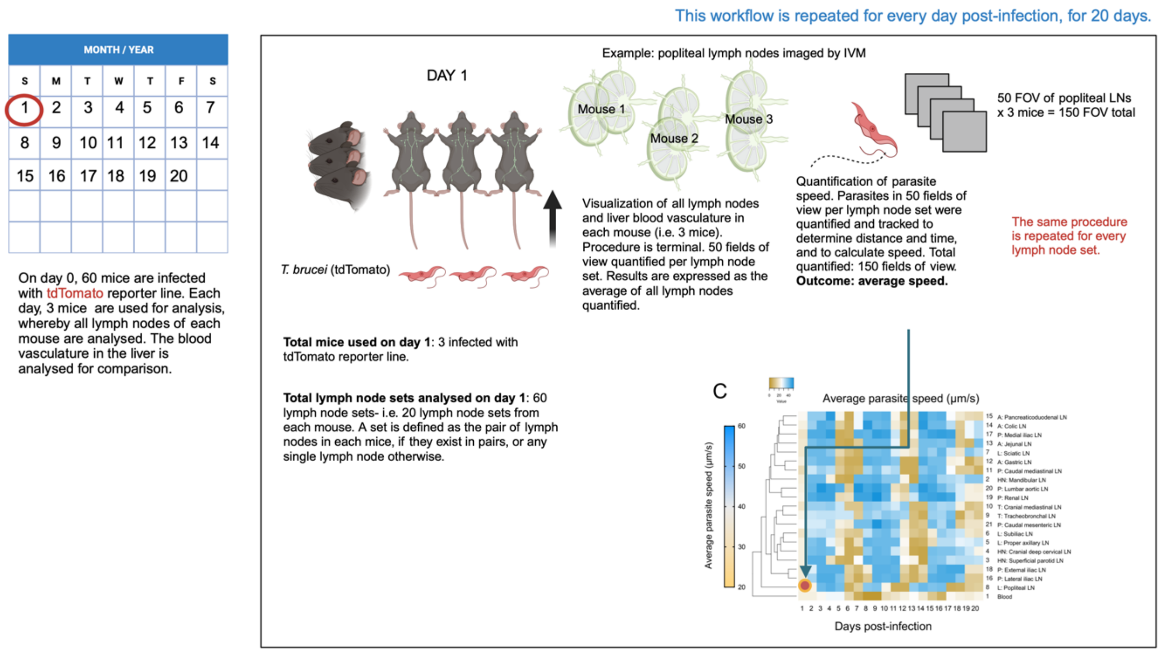


**Fig. S10. Methodological detail corresponding to Figure 3C and 3D.** 60 mice were infected on day 0, with 3000 parasites of the AnTat 1.1E chimeric triple reporter line expressing red-shifted firefly luciferase, tdTomato and Ty1. Parasites were injected intraperitoneally. Every day, starting at day 1 post-infection, 3 mice were used and 20 lymph node sets of each mouse were analysed. The schematic shown in this figure shows the workflow corresponding to day 1 in the popliteal lymph node as an example. Parasites in 50 fields of view of each lymph node set in each mouse were used for quantification, giving a total of 150 fields of view, each corresponding to a time lapse of 20 seconds. A minimum of 100 parasites were quantified in total. The parasite speed was calculated as distance covered over time. This included all forms of motion (i.e. parasites that swim and displace, parasites that tumble, and parasites that do intermediate motion (swim and tumble)). The arrow points to the value that the workflow corresponding to the popliteal lymph node at day 1, leads to. 60 lymph node sets (20 lymph node sets per mouse) are explored each day. Quantifications are the result of averages of 3 mice per day for each lymph node set.

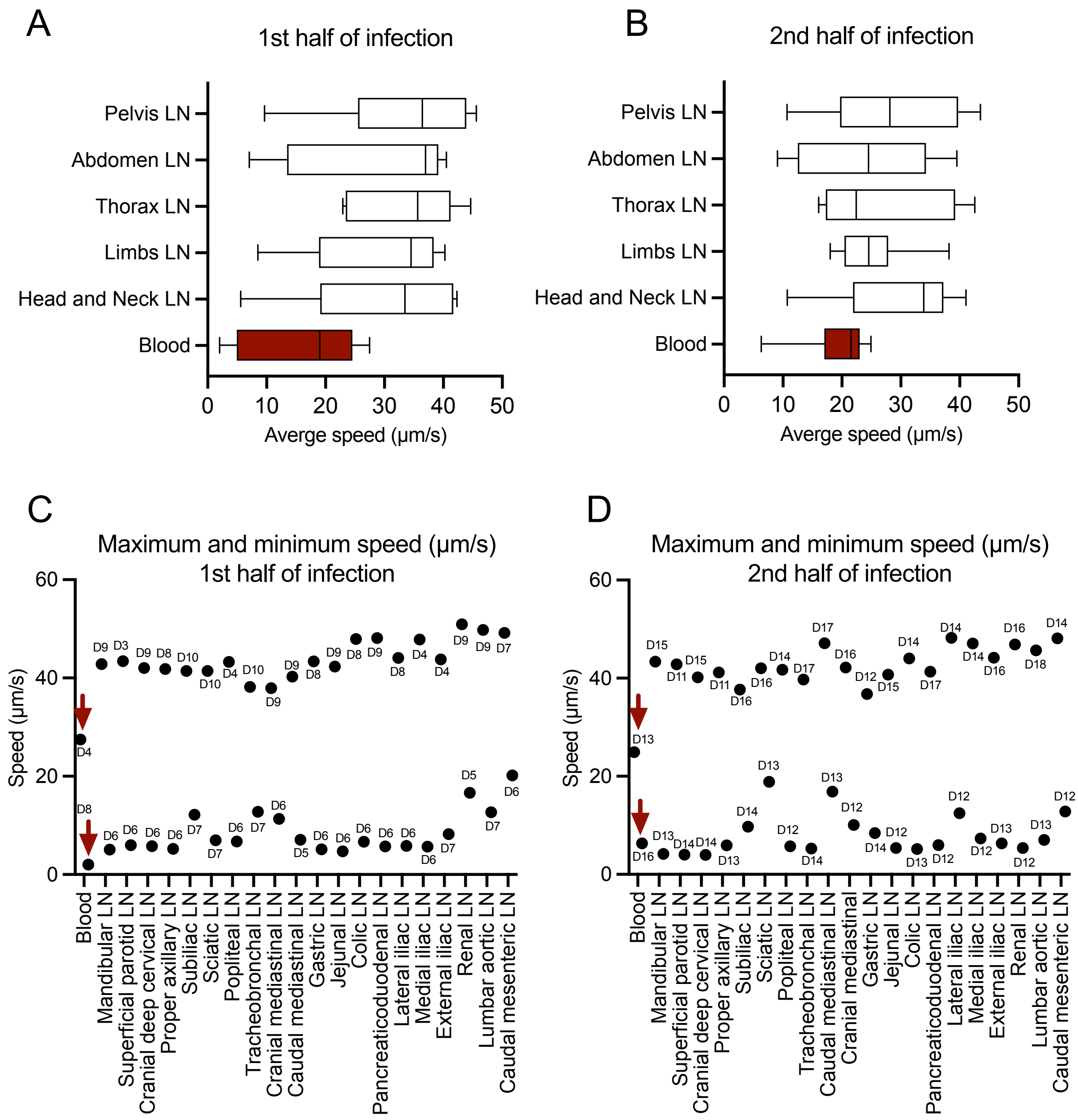


**Fig. S11. The *T. brucei* population in the lymph nodes is behaviourally different to the one in blood.** Changes in speed were infection-time-dependent with drastic variations occurring within a short period of time during both parasitemia waves. **A)** During the first 10 days of infection, parasites in the blood had a median speed of 19.04 µm/s, while the lymph node parasite populations showed a median speed of 36.1 µm/s. **B)** During the last 10 days of infection, parasites in the blood had a median speed of 21.5 µm/s, while the lymph node parasite populations showed a median speed of 30.1 µm/s. **C-D)** Considering all time points, the maximum speed reached by parasites in any lymph node (50.92 µm/s), was significantly higher to the one reached in blood (27.46 µm/s).

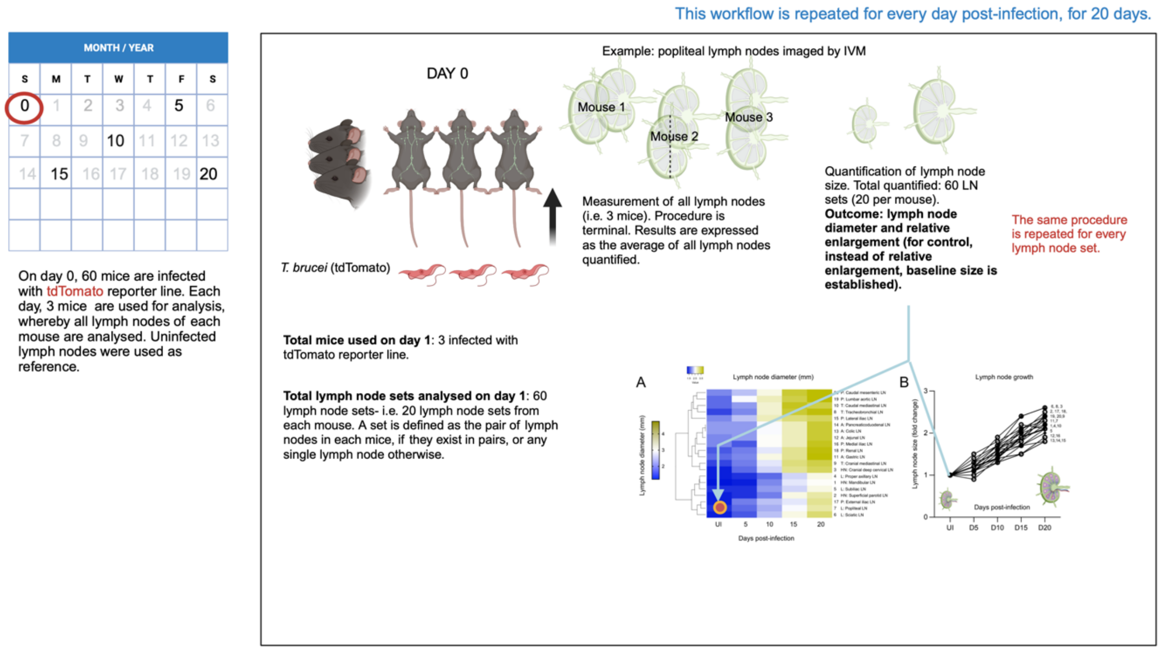


**Fig. S12. Methodological detail corresponding to Figure 5A-5B.** 60 mice were infected on day 0, with 3000 parasites of the AnTat 1.1E chimeric triple reporter line expressing red-shifted firefly luciferase, tdTomato and Ty1. Parasites were injected intraperitoneally. Every day, starting at day 1 post-infection, 3 mice were used and 20 lymph node sets of each mouse were analysed. The schematic shown in this figure shows the workflow corresponding to day 1 in the popliteal lymph node as an example. Lymph node diameter was measured in uninfected controls, and in mice at days 5, 10, 15, and 20 post-infection. Results are expressed as relative growth, compared to uninfected controls. The arrow points to the value that the workflow corresponding to the popliteal lymph node at day 1, leads to. 60 lymph node sets (20 lymph node sets per mouse) are explored each day. Quantifications are the result of averages of 3 mice per day for each lymph node set.

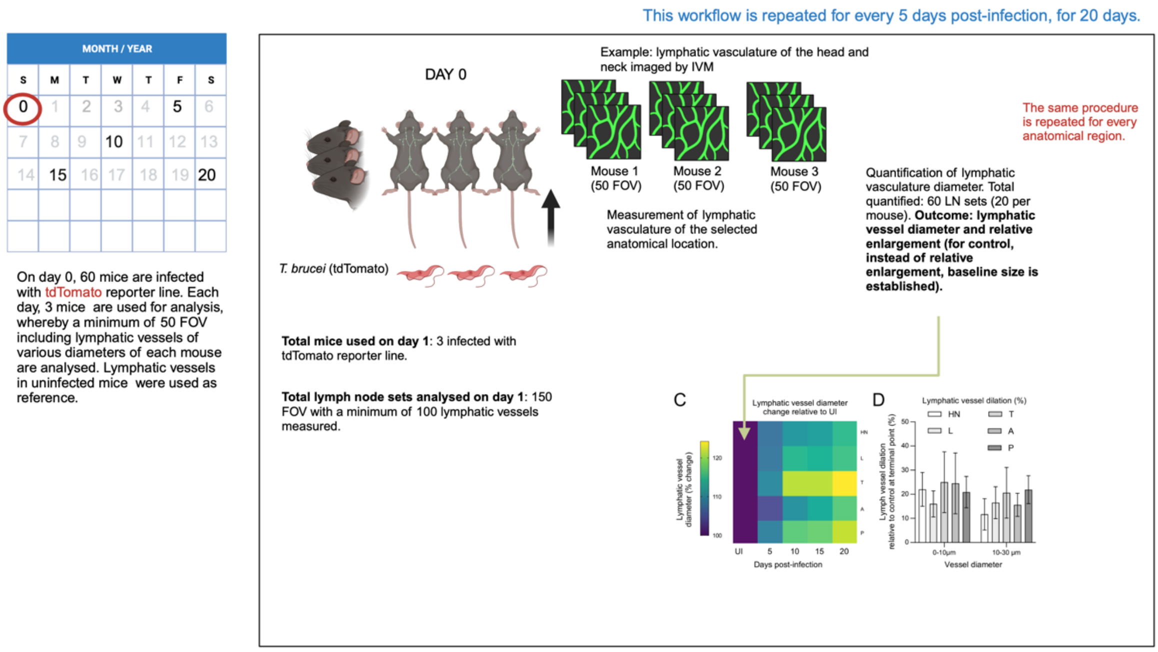


**Fig. S13. Methodological detail corresponding to Figure 5C-5D.** 60 mice were infected on day 0, with 3000 parasites of the AnTat 1.1E chimeric triple reporter line expressing red-shifted firefly luciferase, tdTomato and Ty1. Parasites were injected intraperitoneally. Every day, starting at day 1 post-infection, 3 mice were used and 20 lymph node sets of each mouse were analysed, with vessels in 50 fields of view per mouse being considered. The schematic shown in this figure shows the workflow corresponding to day 1 in the lymphatic vasculature of the head and neck region as an example. Lymphatic vessel diameter was measured in uninfected controls, and in mice at days 5, 10, 15, and 20 post-infection. Results are expressed as relative dilation, compared to uninfected controls. The arrow points to the value that the workflow corresponding to the head and neck region in uninfected mice, leads to. 60 lymph node sets (20 lymph node sets per mouse) are explored each day. Quantifications are the result of averages of 3 mice per day for each lymph node set.

Biology Open • Supplementary information

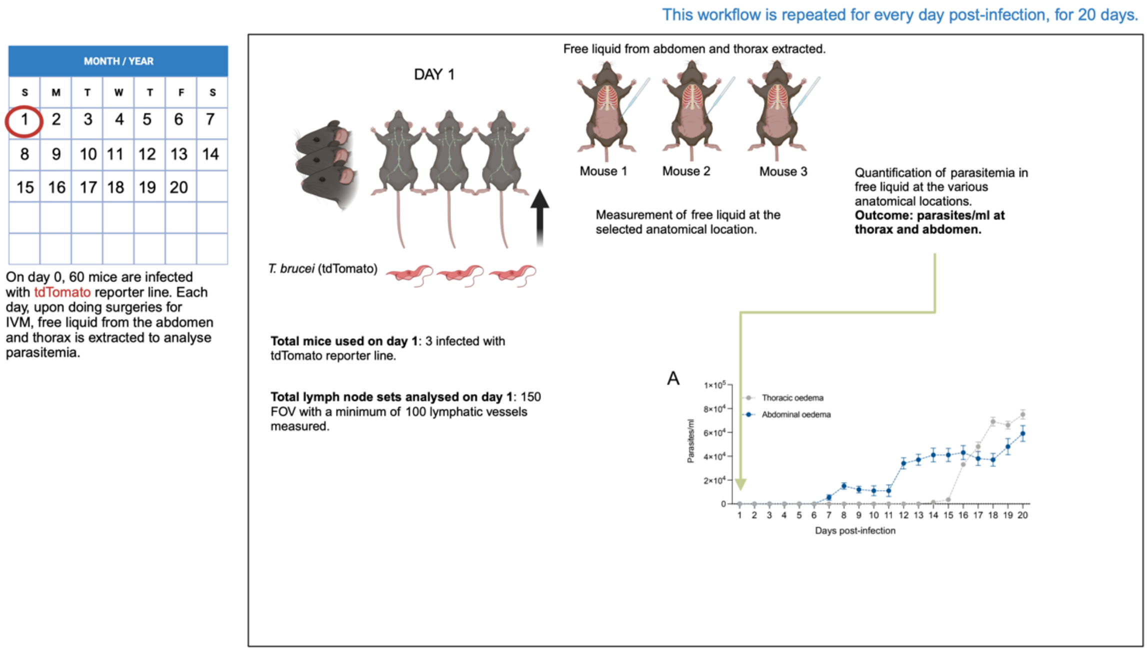


**Fig. S14. Methodological detail corresponding to Figure 6A.** 60 mice were infected on day 0, with 3000 parasites of the AnTat 1.1E chimeric triple reporter line expressing red-shifted firefly luciferase, tdTomato and Ty1. Parasites were injected intraperitoneally. Every day, starting at day 1 post-infection, 3 mice were used, and free fluid in the thoracic and abdominal cavities was collected, and parasites were quantified by hemocytometer. The schematic shown in this figure shows the workflow corresponding to day 1. The arrow points to the value that the workflow corresponding to day 1. Quantifications are the result of averages of 3 mice per day.

Biology Open • Supplementary information

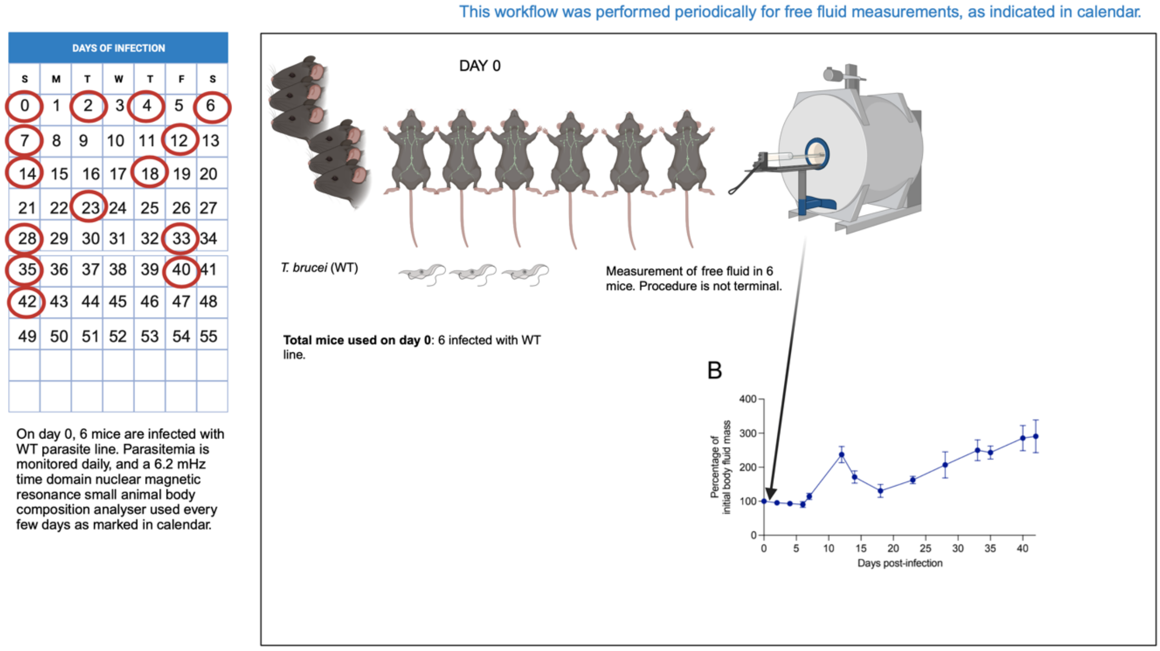


**Fig. S15. Methodological detail corresponding to Figure 6B.** 6 mice were infected on day 0, with 3000 parasites of the AnTat 1.1E WT line. Parasites were injected intraperitoneally. Every day, starting at day 1 post-infection, all mice were analysed using magnetic resonance to detect body fluid mass. This is a non-invasive procedure that was done periodically as shown in the calendar schematic.  The arrow points to the value that the workflow corresponding to day 1. Quantifications are the result of averages of 6 mice per day.

Biology Open • Supplementary information

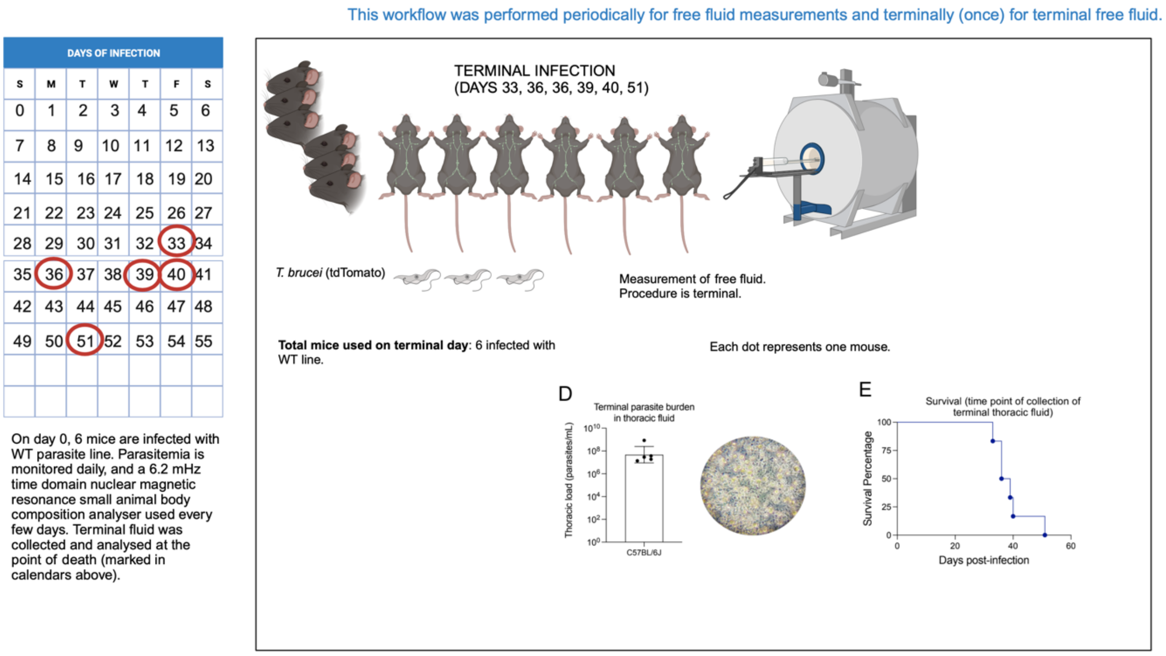


**Fig. S16. Methodological detail corresponding to Figure 6D-6E.** 6 mice were infected on day 0, with 3000 parasites of the AnTat 1.1E WT line. Parasites were injected intraperitoneally. Toward the end of the survival period, free fluid was measured by MRI, and the fluid was collected by thoracic aspiration. Parasites were quantified using a hameocytometer, and expressed as parasites/ml. Figure 6E shows the survival of all mice, indicating the point at which fluid was measured and parasite burden calculated. This is the result of 6 mice.

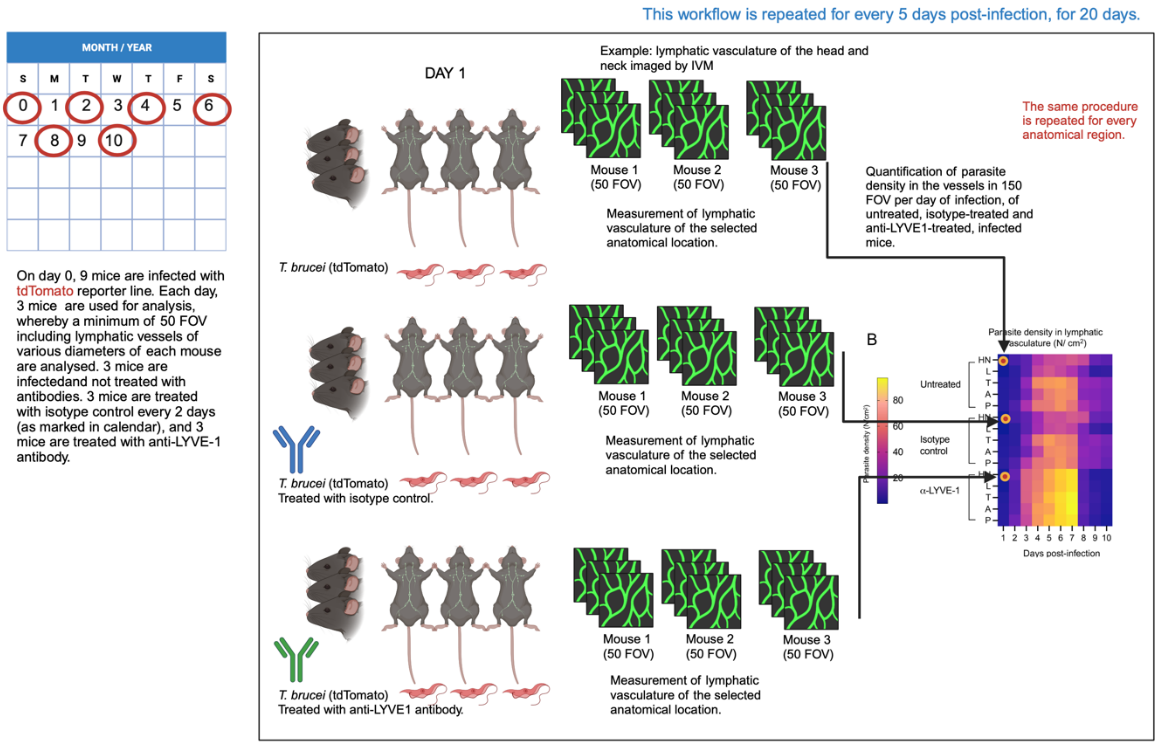


**Fig. S17. Methodological detail corresponding to Figure 6D-6E.** 9 mice were infected on day 0, with 3000 parasites of the AnTat 1.1E chimeric triple reporter line expressing red-shifted firefly luciferase, tdTomato and Ty1. Parasites were injected intraperitoneally. Every day, starting at day 1 post-infection, the lymphatic vasculature of the head and neck, lymph, thorax, abdomen and pelvis was analysed. The schematic shown in this figure shows the workflow corresponding to day 1 in the lymphatic vasculature of the head and neck region as an example. Parasite density in lymphatic vessels is measured in mice at days 1-10 post-infection. 3 groups of mice are analysed: control mice, mice treated with an isotype control, and mice treated with anti-LYVE1 blocking antibody. The arrow points to the value that the workflow corresponding to the head and neck region in each group of mice at day 1 post-infection, leads to. Quantifications are the result of averages of 3 mice per day for each group.

**Table S1.** Parasite density (related to Figure 1B)

Available for download at
https://journals.biologists.com/bio/article-lookup/doi/10.1242/bio.059992#supplementary-data

**Table S2.** Parasite density (related to Figure 1C)

Available for download at
https://journals.biologists.com/bio/article-lookup/doi/10.1242/bio.059992#supplementary-data

**Table S3.** Parasite survival (related to Figure 1E and 1F)

Available for download at
https://journals.biologists.com/bio/article-lookup/doi/10.1242/bio.059992#supplementary-data

**Table S4.** Percentage of 1K1N (related to Figure 2A and 2B)

Available for download at
https://journals.biologists.com/bio/article-lookup/doi/10.1242/bio.059992#supplementary-data

**Table S5.** Percentage of slenders (related to Figure 2D and 2E)

Available for download at
https://journals.biologists.com/bio/article-lookup/doi/10.1242/bio.059992#supplementary-data

**Table S6.** Parasite width (related to Figure S2A)

Available for download at
https://journals.biologists.com/bio/article-lookup/doi/10.1242/bio.059992#supplementary-data

**Table S7.** Parasite length (related to Figure S2B)

Available for download at
https://journals.biologists.com/bio/article-lookup/doi/10.1242/bio.059992#supplementary-data

**Table S8.** Parasite area (related to Figure 3A-3B)

Available for download at
https://journals.biologists.com/bio/article-lookup/doi/10.1242/bio.059992#supplementary-data

**Table S9.** Parasite speed (related to Figure 3C-3D)

Available for download at
https://journals.biologists.com/bio/article-lookup/doi/10.1242/bio.059992#supplementary-data

**Table S10.** Normalized values used for Figures 4A and 4B

Available for download at
https://journals.biologists.com/bio/article-lookup/doi/10.1242/bio.059992#supplementary-data

**Table S11.** MAD scores for Figure 4C

Available for download at
https://journals.biologists.com/bio/article-lookup/doi/10.1242/bio.059992#supplementary-data

**Table S12.** Lymph node growth for Figure 5A and 5B

Available for download at
https://journals.biologists.com/bio/article-lookup/doi/10.1242/bio.059992#supplementary-data

**Table S13.** Lymphatic vessel dilation for Figure 5C,D,E

Available for download at
https://journals.biologists.com/bio/article-lookup/doi/10.1242/bio.059992#supplementary-data

Biology Open • Supplementary information

**Table S14.** Parasitemia in oedemas and free fluid: Figure 6

Available for download at
https://journals.biologists.com/bio/article-lookup/doi/10.1242/bio.059992#supplementary-data

**Table S15.** LYVE-1 blocking effect on parasite density in lymphatic vessels - Figure 7A- 7C

Available for download at
https://journals.biologists.com/bio/article-lookup/doi/10.1242/bio.059992#supplementary-data

**Table 16.** LYVE-1 blocking effect on parasite density in blood vessels - Figure 7D-7F

Available for download at
https://journals.biologists.com/bio/article-lookup/doi/10.1242/bio.059992#supplementary-data
